# Thioamide-based fluorescent sensors for dipeptidyl peptidase 4†

**DOI:** 10.1039/d4cc03309d

**Published:** 2024-10-23

**Authors:** Hoang Anh T. Phan, Yanan Chang, Samuel A. Eaton, E. James Petersson

**Affiliations:** a Department of Chemistry, School of Arts and Sciences, University of Pennsylvania 231 South 34th Street Philadelphia Pennsylvania 19104 USA ejpetersson@sas.upenn.edu; b Department of Biochemistry and Biophysics, Perelman School of Medicine, University of Pennsylvania 421 Curie Boulevard Philadelphia PA 19104 USA

## Abstract

Dipeptidyl peptidase 4 (DPP-4) is a promising biomarker for cancer and metabolic diseases. We demonstrate the design of novel fluorescent DPP-4 probes based on the protease's native substrates using a thioamide as a quencher for measuring *in vitro* kinetics, inhibition with sitagliptin, and DPP-4 activity in saliva samples.

## Abstract 2

Thioamide sensors detect DPP-4 activity in human saliva.

## Graphical abstract

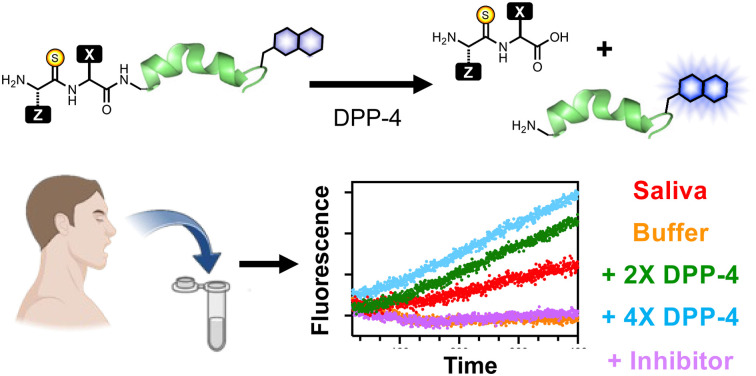

Dipeptidyl peptidase 4 (DPP-4) is a serine protease that preferably cleaves at a proline or an alanine at the second amino acid of its substrates, liberating dipeptides.^1^ Interestingly, DPP-4 is a multifunctional protein also known as CD26, which serves as T cell costimulatory receptor in addition to its enzymatic activity.^2^ When DPP-4 was first discovered by Hopsu-Havu and Glenner in 1966, it was initially called glycyl-proline naphthylamidase due to the fact that it could cleave the substrate glycyl-dl-prolyl-β-naphthylamide (GP-βNA).^3^ DPP-4 can inactivate peptides whose N-termini are important for biological function or cleave peptides to generate new ones.^4,5^

Among the natural substrates of DPP-4 are the peptides from the pituitary adenylate cyclase-activating polypeptide (PACAP)/glucagon family such as glucagon-like polypeptide 1 (GLP-1) and gastric inhibitory polypeptide (GIP), which are important in regulating glucose homeostasis.^4,6^ GLP-1 stimulates insulin release, but it can be rapidly cleaved by DPP-4 with a half-life of less than 2 minutes. There have been major advances in DPP-4-based therapeutics for treatment of diabetes, especially in creating stabilized GLP-1 analogs and DPP-4 inhibitors such as sitagliptin (Januvia).^7–10^ Beyond GLP-1, DPP-4 cleaves a diverse repertoire of bioactive peptides, such as vasoactive intestinal peptide (VIP), substance P, pancreatic polypeptide (PP), peptide YY (PYY), and neuropeptide Y (NPY). DPP-4 is expressed in different tissues as either an anchored transmembrane protease or a soluble circulating one, and its anomalous activity has also been linked to cancer (solid tumors), hematological malignancies, immune diseases, and infectious diseases.^4,5^ Studies have identified DPP-4 as a promising biomarker for early diagnosis and treatment of diseases such as lung diseases, diabetic kidney disease,^11^ rheumatoid arthritis,^12^ post-kidney transplant tubulitis,^12^ and liver disease.^13^ There is thus a need to develop probes to monitor DPP-4 activity real-time.

Common chromogenic substrates for DPP-4 include GP-βNA,^3^ Ala-Pro-7-amino-4-trifluoromethyl-coumarin (AP-AFC), and Gly-Pro-7-amido-4-methylcoumarin (GP-AMC). Many DPP-4 probes utilize a pro-fluorophore Förster resonance energy transfer (FRET), photoinduced electron transfer (PeT), or intramolecular charge transfer (ICT) approach where the fluorophore is quenched when conjugated to a short recognition dipeptide (*e.g.*, Gly-Pro; Ala-Pro), and can return to its highly emissive state upon cleavage by the protease.^14–17^ These strategies can perturb and compromise the recognition sequences, thereby preventing accurate reporting of proteolysis. For instance, in ICT-based probes GP-βNA, AP-AFC, and GP-AMC, the chromophore is attached directly to the cleavage site so no information can be gained on the C terminal side (called the prime side) of the cleavage site. Even the N-terminal (non-prime) side information can be compromised. A study showed that P1 substitution in the P2–P1-AMC probes did not reflect mutation effects observed in the full-length peptide substrates of DPP-4.^18^ Additionally, an approach utilizing FRET pairs, that require labeling on either side of the scissile bond, is not compatible with monitoring aminopeptidases or carboxypeptidases where recognition of the amino acid at the substrate terminus is essential.

Our laboratory has previously shown that thioamides can quench donor fluorophores such as tryptophan, tyrosine, fluorescein, acridonylalanine, and 7-methoxycoumarin, serving as a minimally perturbing probe for protein dynamics and proteolysis.^19–23^ For the general design, we typically incorporate a thioamide quencher on the opposite of the scissile bond from the fluorophore; upon cleavage by proteolysis, these two will be separated, resulting in a turn-on of fluorescence that can be monitored in real time (Fig. 1A). We have systematically studied thioamide positional effects on our fluorescence sensor probes for cysteine proteases (papain, cathepsins L, V, K, B, and S) and serine proteases (trypsin, chymotrypsin, and kallikrein), identifying trends in perturbing and non-perturbing sites for thioamide labeling.^24^ We have also tested thioamide peptides to determine whether they interfere with the cleavage of other substrates (showing that they bind at the active site).^21–23,25^ This initial screening allows us to use thioamide in one of three ways at a given site (Fig. 1B): (1) non-perturbing, interfering sites can be used for fluorescence protease sensors,^20^ (2) perturbing, non-interfering sites can be used for *in vivo* therapeutic or imaging peptides (*e.g.*, thioamide-stabilized therapeutic GLP-1 or NPY-based cancer imaging probes),^21,25^ (3) perturbing, interfering sites can be used for selective protease inhibitors (*e.g.*, thioamide cathepsin L inhibitors).^23^ Herein, we follow this workflow to develop thioamide peptide probes for DPP-4 based on its native substrates. Our probes, containing 7-methoxycoumarin-4-yl-alanine (Mcm or μ, Fig. 2A) as a fluorophore, can effectively report real-time DPP-4 activity in *in vitro* steady-state assays, inhibition assays, and saliva samples. We thus highlight the value of thioamides for the minimally perturbing design of substrate-based protease probes.

**Fig. 1:**
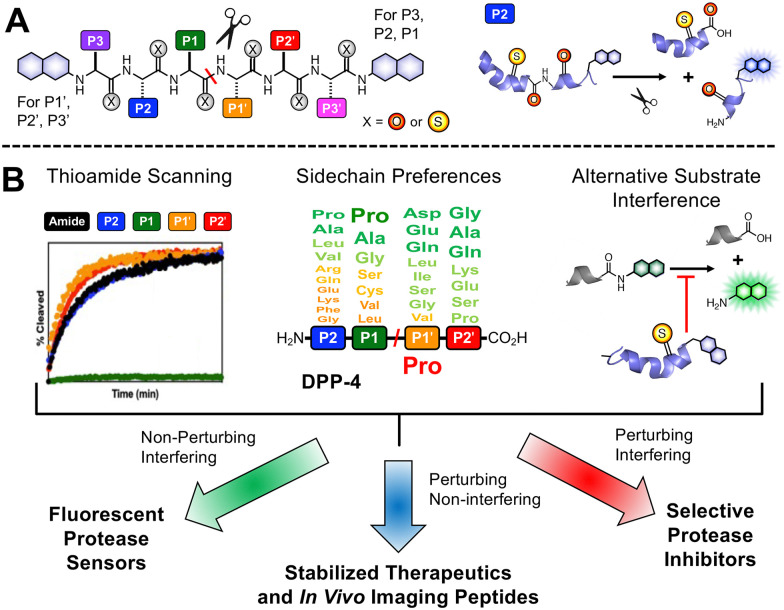
Thioamide applications in protease sensors, peptide stabilizers, and inhibitors. (A) General design of thioamide protease sensor probes. Thioamides can quench a N- or C-terminally tagged fluorophore when they are in proximity; upon cleavage, there will be a turn on in fluorescent signal. Cleavage of a P2 thioamide peptide is shown as an example. (B) Information on thioamide effects on proteolysis rates from sensor probe assays, substrate sidechain preferences from literature studies and genetic data, and measurements of interference in cleavage of alternative substrates, can be leveraged for development of substrate-based thioamide probes. In this work, non-perturbing thioamide labeling can be used for the design of fluorescent protease sensors for DPP-4.

**Fig. 2:**
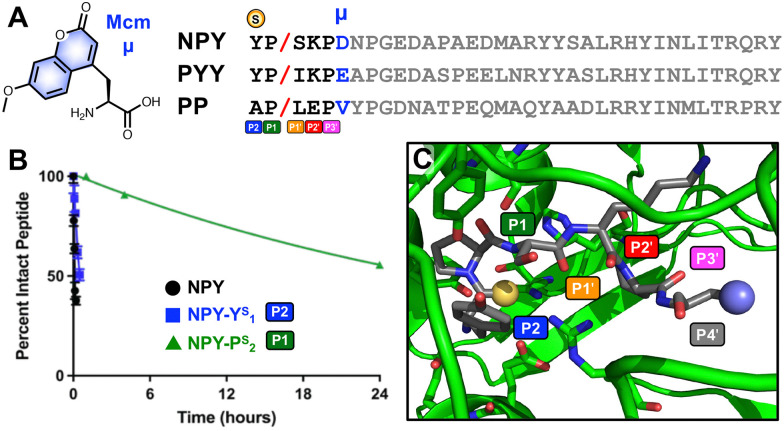
Design of substrate-based thioamide probes for DPP-4. (A) Sequences of DPP-4 substrates neuropeptide Y (NPY), peptide YY (PYY), and pancreatic polypeptide (PP), with N-terminal residues P2–P3′ highlighted and the P4′ position in blue, where the donor fluorophore Mcm (μ) is placed. (B) Cleavage by DPP-4 of full-length NPY, NPY-Y^S^_1_ (P2 thioamide), and NPY-P^S^_2_ (P1 thioamide) monitored by HPLC. (C) X-ray structure of DPP-4 (green) with a fragment of NPY bound (grey), showing the P2–P4′ positions with the P2 thioamide shown as a yellow sphere and the Mcm sidechain shown as a blue sphere.

We based our first peptide probe design on NPY since it is known to be an excellent substrate of DPP-4.^4,26^ NPY is a natural 36-amino-acid neuromodulator that is abundant in the brain as well as in the peripheral nervous system.^27^ NPY can modulate NPY receptors, which have been implicated in many diseases such as metabolic diseases, obesity, pain, cancer, and cardiovascular regulation. DPP-4 typically cleaves full-length NPY at Pro_2_ at its N-terminus, resulting in NPY_3-36_.^4,5,26^ DPP-4 cleavage modulates NPY receptor selectivity since the NPY_3-36_ fragment is no longer active toward the NPY Y1 receptor (with vasoconstrictive properties), yet has a strong affinity toward NPY Y2 and Y5 receptors (as a vascular growth factor).^4,5^

Since DPP-4 cleaves the NPY peptides at Pro_2_, we have only two options for thioamide placement, at the first tyrosine residue Y_1_ (P2 position) or at the second proline P_2_ (P1 position). As a preliminary assessment, we synthesized the all-amide full-length NPY peptide, along with its P1 thioamide analog (NPY-P^S^_2_) and its P2 thioamide analog (NPY-Y^S^_1_), to evaluate which thioamide position on NPY would significantly perturb recognition by DPP-4. In these assays, cleavage of the peptides was monitored high-performance liquid chromatography (HPLC), and fragment identities were confirmed by matrix-assisted laser desorption ionization mass spectrometry (MALDI MS) (Fig. 2B, primary data are shown in Fig. S11–S13, ESI†). The P2 thioamide analog, NPY-Y^S^_1_, cleaved 6.5 times slower than the all-amide counterpart, while the P1 thioamide, NPY-P^S^_2_, cleaved 372 times slower. This is in contrast to our results with GLP-1 and GIP thioamidation, in which both P2 and P1 thioamides significantly retarded proteolysis.^25^ Here, our data show that the P2 position is less perturbing for proteolysis, possibly because it is a proline residue, and that we should place the thioamide at the P2 position to create a quenching effect as desired while still being responsive to DPP-4 proteolysis.

For our probe design, we chose Mcm/μ as the fluorophore for the following reasons: (1) it is only slightly larger than Trp; (2) it is commercially available as Fmoc-μ-OH; (3) it can be easily incorporated into the peptide probes *via* solid phase peptide synthesis, and (4) it can be quenched by thioamides. Our lab also has previously successfully utilized Mcm-tagged peptides as proteolysis probes for different serine-, cysteine-, carboxyl-, and metallo-proteases.^20–22,24^ Since μ is quenched by thioamides *via* a PeT mechanism, they must be in close proximity.^19^ Based on our results with NPY-P^S^_2_ and NPY-Y^S^_1_, as well as an analysis of the crystal structure of DPP-4 with a fragment of NPY bound (PDB ID: 1r9n),^28^ we decided to design a hexapeptide probe, with the thioamide at P2 position (*i.e.* the N terminus) and the fluorophore μ at the P4′ position, since the DPP-4 active site is relatively open there while it makes close contacts with the P1′, P2′, and P3′ amino acids (Fig. 2C). P2 and P4′ are 5 residues apart, predicted to give sufficient quenching based on our studies of thioamide PeT quenching.^19^

First, we synthesized probe analogs of NPY, with the sequence of YPSKPμ (NPY_1–5_). For comparison, we synthesized both the all-amide and P2 thioamide version of this peptide, YP/SKPμ and Y^S^P/SKPμ, where the / indicates the DPP-4 cleavage site (Fig. 2A). As a demonstration that we could study mutations at prime sites, we also made a probe based on the NPY mutation Ser_3_-to-Leu (NPY_1−5_L_3_: YPLKPμ). The same design was applied to two other natural substrates from the pancreatic polypeptide family, where we synthesized and tested the hexapeptide probes based on the first 5 N-terminal amino acids of PYY (PYY_1–5_: YPIKPμ) and PP (PP_1–5_: APLEPμ). NPY_1–5_ NMR data indicate that the probes are unstructured (Fig. S25–S27, ESI†).

Each probe was incubated with DPP-4 and fluorescence intensity was monitored over time (*λ*_ex_ = 325 nm; *λ*_em_ = 390 nm). Significantly, our μ-labeled NPY P2 thioamide sensor, Y^s^PSKPμ, was cleaved by DPP-4 with similar kinetics (Fig. 3A, *t*_1/2_ = 26 min) to the full-length NPY P2 thioamide, NPY-P^S^_2_ (*t*_1/2_ = 30 min). Interestingly, we observed a fluorescence turn-on even without a thioamide, which is presumably due to quenching by the N-terminal Tyr residue. In fact, for both NPY probes (Fig. 3A and B) and the PYY probe (Fig. 3C), we observed the synergic effects of both Tyr and thioamide quenching. In the case of the PP-based probe, since there was no tyrosine, the thiopeptide showed its full utility as the all-amide APLEPμ had no fluorescence quenching, while the thioamide version A^S^PLEPμ showed good fluorescence turn-on (Fig. 3D). To ensure that the fluorescence assays truly reflect protease cleavage, HPLC and MALDI were used to confirm cleavage product identities (Fig. S14–S17 and Tables S4–S7, ESI†). The P1′ mutated NPY_1–5_L_3_ probe, Y^S^PLKPμ, is cleaved 2-fold slower than the native NPY_1–5_ probe, Y^S^PSKPμ, and PYY_1–5_, which also differs at the P1′ site (Y^S^PIKPμ), is cleaved 3-fold slower than NPY_1–5_. These data demonstrate that we can create probes to explore the effects of mutations on the prime sites of a substrate, which could not be achieved with commercially available probes such as GP-AMC.

**Fig. 3:**
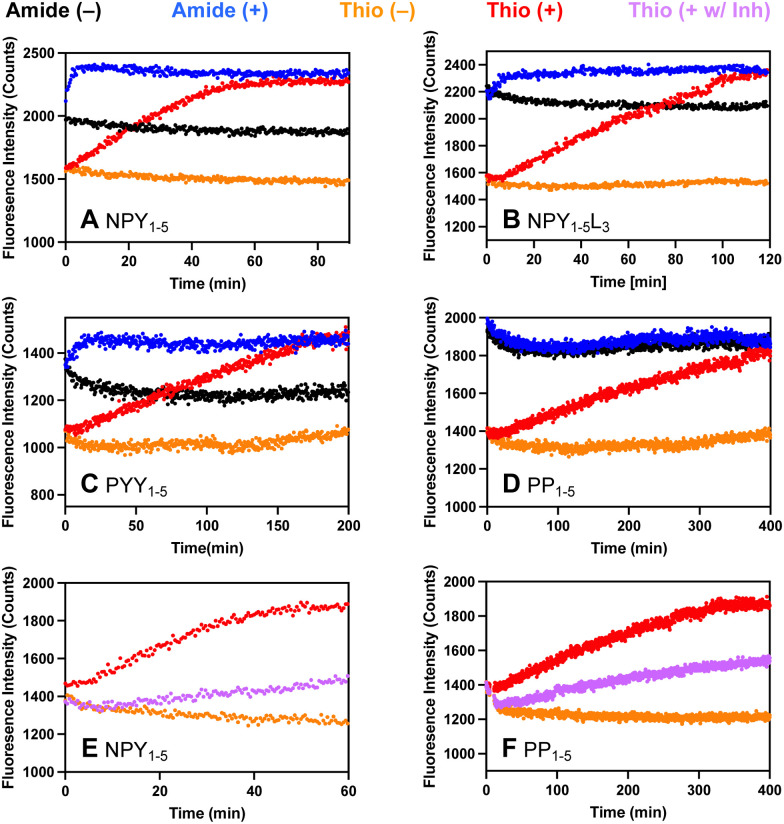
DPP-4 proteolysis probes based on native substrates from the pancreatic polypeptide family. (A)–(F) Fluorescence turn-on occurs upon DPP-4 cleavage due to relief of thioamide quenching and/or Tyr quenching. The (−) indicates the absence of protease and the (+) indicates the presence of protease. For thioamide NPY_1–5_ and PP_1–5_ peptides, (E) and (F) assays were also done with the protease and 50 nM inhibitor sitagliptin (+w/Inh). The fluorescence was monitored as a function of time at 390 nm with excitation at 325 nm on a Tecan Spark plate reader. All traces show the average of three replicates.

To ensure that our probes are responsive to the presence of inhibitors, inhibition studies were done with 50 nM sitagliptin, which is a selective inhibitor of DPP-4 with an IC_50_ between 18–87 nM, depending on the experiment.^10,29^ In all cases, we observed inhibition by sitagliptin with our probes, as indicated by a decrease in fluorescence signal in the presence of 50 nM sitagliptin consistent with ∼50% inhibition (NPY and PP probe data shown in Fig. 3E and F; NPY-L_3_ and YY probe data show in Fig. S19, ESI†). At higher concentrations of sitagliptin, we observe complete inhibition (Fig. S20, ESI†). This shows the potential of using our probes in inhibitor screening in biochemical assays, which is an important application for drug development and mechanistic studies.

We also wished to demonstrate that our probes could be used as a tool to quickly detect DPP-4 activity in biological samples. For instance, DPP-4 activity in saliva has been associated with diseases such as periodontitis and the presence of *Porphyromonas gingivalis*, Sjögren's syndrome, and oral cancers.^30–32^ Herein, following a very simple protocol, we directly added our thioamide probes to human saliva, then measured fluorescence changes with a plate reader (Fig. 4A). For our NPY- and PYY-based probes, we observed an increase in fluorescence in the presence of saliva in the first two hours of incubation (NPY_1–5_ probe data shown in Fig. 4B, NPY_1–5_L_3_ and PYY_1–5_ probe data shown in Fig. S22, ESI†). For PP_1–5_, a slower increase in fluorescence was observed (Fig. 4C), consistent with slower rates in the DPP-4 only assay. The fluorescence increases could be completely blocked by sitagliptin, showing that they are highly specific for DPP-4 (Fig. 4 and Fig. S23, ESI†). To approximate saliva samples from cancer patients, where DPP-4 activity levels are 2–4 fold higher than in controls, we added DPP-4 to the saliva and found that we could easily detect these differences (Fig. 4B and C). These results show the potential for our probes to measure DPP-4 in biological samples.

**Fig. 4:**
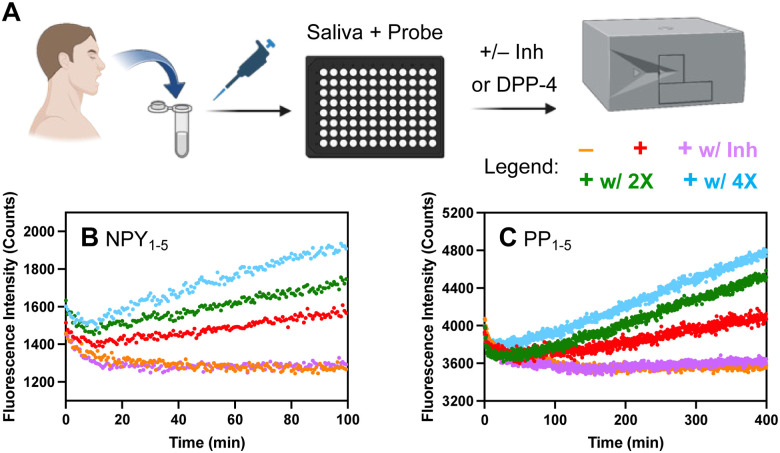
DPP-4 detection in human saliva. (A) Assay workflow, created with Biorender.com. (B) and (C) For each assay, 25 μL of human saliva (+), saliva with 250 ng L^−1^ (+w/2×) or 500 ng L^−1^ (+w/4×) additional DPP-4, PBS (−), or saliva with 500 nM sitagliptin (+w/Inh) was added to 25 μL of 5 μM NPY_1–5_ or 15 μM PP_1–5_ thioamide probe. The fluorescence was monitored as a function of time at 390 nm with excitation at 325 nm on a Tecan Spark plate reader. Traces show the average of three replicates.

Here, we have demonstrated that we can create thioamide probes to detect DPP-4 activity using substrate-based probes that not only include the dipeptide recognition motif, but also native sequences on the primed side of the scissile bond – something that is not featured in many of the existing probes. This close mimicking of the native substrate allows our probes to report DPP-4 activity as faithfully as possible. Given the promising results from the human saliva assays, we will further test with actual patient samples. We will take advantage of the thioamide's ability to quench other fluorophores^19^ to make cellular or *in vivo* imaging probes with red-shifted dyes, as in many recent cancer imaging probes.^33,34^

The manuscript was written by H. A. T. P. and E. J. P., with input from all authors. H. A. T. P. designed all experiments and H. A. T. P. and Y. C. performed most experiments. S. A. E. synthesized full-length NPY constructs.

This research was supported by a grant from the National Science Foundation to E. J. P. (NSF CHE-2203909). The MALDI MS was acquired through a National Institutes of Health instrumentation grant (NIH S10-OD030460).

## Data availability

The data supporting this article have been included as part of the ESI.†

## Conflicts of interest

There are no conflicts to declare.

## Supplementary Material

CC-060-D4CC03309D-s001
