# Supplementary material for: Thioamide-based fluorescent sensors for dipeptidyl peptidase 4
Source: Chem Commun (Camb). 2024 Oct 23;60(89):13075–8. doi: 10.1039/d4cc03309d (PMC11497070; doi:10.1039/d4cc03309d)
Supplement: CC-060-D4CC03309D-s001 [file CC-060-D4CC03309D-s001.pdf]

Supplementary Information (SI) for ChemComm.
This journal is © The Royal Society of Chemistry 2024

Thioamide-based Fluorescent Sensors for Dipeptidyl Peptidase 4

Hoang Anh T. Phan,[a] Yanan Chang,[a] Samuel A. Eaton,[a] E. James Petersson [a]

[a] *Department of Chemistry, University of Pennsylvania, Philadelphia, Pennsylvania 19104, USA*

*Email: ejpetersson@sas.upenn.edu

Table of Contents

1. General Information 2
2. Peptide Synthesis, Purification, and Characterization 3
3. DPP-4 Proteolysis Assays with Full-Length NPY Peptides (HPLC) 17
4. DPP-4 Proteolysis Assays (Steady State Assays) 21
5. Sitagliptin Inhibition of DPP-4 26
6. Human Saliva Assays 29
7. DPP-4 Inhibition with Sitagliptin in Human Saliva 31
8. Human Saliva Doped with Additional DPP-4 33
9. Nuclear Magnetic Resonance (NMR) 34
10. References 36

1. General Information

Fluorenylmethoxycarbonyl-β-(7-methoxycoumarin-4-yl)-Ala-OH were purchased from Bachem (Torrance, CA, USA). All other fluorenylmethoxycarbonyl (Fmoc) protected amino acids were purchased from Novabiochem (currently EMD Millipore, MilliporeSigma; Burlington, MA, USA). (7-Azabenzotriazol-1-yloxy)tripyrrolidino-phosphonium hexafluorophosphate (PyAOP) was purchased from Chem-Impex (Wood Dale, IL, USA). Piperidine, *N*,*N*-diisopropylethylamine (DIPEA), dipeptidyl Peptidase IV human (DPP-4, recombinant, expressed in Sf9 cells), and sitagliptin (DPP-4 specific inhibitor, ≥98% by HPLC; SML3205) were purchased from Sigma-Aldrich (St. Louis, MO, USA). Fluorescent H-Gly-Pro-AMC (DPP-4 substrate, lyophilized, ≥95% by HPLC) was purchased from AnaSpec. Pooled human saliva (IRHUSL5ML) was purchased from Innovative Research (Novi, MI, USA). All other reagents were purchased from Fisher Scientific (Pittsburgh, PA, USA) unless specified otherwise. Milli-Q filtered (18 MΩ) water was used for all solutions (EMD Millipore). Peptides were purified with an Agilent 1260 Infinity II Preparative HPLC system and analyzed with an Agilent 1260 Infinity II Analytical HPLC system (Santa Clara, CA, USA). Peptide mass spectrometry was collected with a Bruker Ultraflex III matrix-assisted laser desorption ionization mass spectrometer (MALDI MS), Bruker MicrofleX (MALDI-TOF MS), or Bruker RapifleX (MALDI-TOF/TOF) (Billerica, MA, USA). Time-course UV-Vis absorbance and fluorescence data were obtained with a Tecan Infinite® M1000 PRO plate reader (Männedorf, Switzerland). The NMR spectra was collected with AVANCE NEO 600 MHz NMR.

2. Peptide Synthesis, Purification, and Characterization

Synthesis of Thioamino acid Precursors. N_α-Fmoc-L-thiotyrosine(tBu)-nitrobenzotriazolide, N_α-Fmoc-L-thioproline-nitrobenzotriazolide, and N_α-Fmoc-L-thioalanine-nitrobenzotriazolide were synthesized and characterized using previously published procedures by our laboratory.[1, 2]

Peptide Synthesis of Full-length NPY$_{1\text{-}36}$ Peptides. NPY$_{1\text{-}36}$ was purchased from Genscript. Fmoc-protected NPY$_{3\text{-}36}$ (Fmoc-SKPDNPGEDAPAEDMARYYSALRHYINLITRQRY; molecular weight = 4233.66 g/mol) was purchased on resin from GenScript. Each peptide was manually synthesized on a 10 µmol scale. The resin was initially added to a dry reaction vessel (RV) and swelled in 2 mL dimethylformamide (DMF) for 30 min with magnetic stirring. After washing and swelling, the resin was incubated with 1 mL of 20% piperidine solution in DMF for 20 mins under stirring for deprotection. The same deprotection procedure was followed for all the subsequent standard amino acids. Between each reaction, the resin was washed extensively with adequate DMF, DCM, and DMF. For a typical 45-minute coupling reaction, 5 equiv. of the standard amino acid and 5 equiv. of PyAOP was dissolved in 1 mL DMF, and added to the RV, with an addition of 10 equiv. of DIPEA. Thioamides were coupled using a modified procedure: for the P1 thioamide peptide (NPY$_{1\text{-}36}$-P$^{S}_{2}$) and P2 thioamide analog (NPY$_{1\text{-}36}$-Y$^{S}_{1}$), 3 equiv. of the thioamide precursor was dissolved in 1 mL of dry dichloromethane (DCM) with 6 equiv. DIPEA and stirred for 30 minutes. This procedure was repeated prior to deprotection to ensure efficient incorporation of the thioaminoacid onto the peptide chains. For the deprotection of thioamides, 1 mL of 2% DBU (1,8-diazabicyclo(5.4.0)undec-7-ene) in DMF was added to the RV and reacted three times for 2 min each, with extensive washing with DMF and DCM between each deprotection step.

Peptide Synthesis of Short NPY-based Fluorescent Probes. Each peptide was manually synthesized on a 25 or 100 μmol scale on 2-chlorotrityl resin based on our established protocols.[3] For a typical synthesis, 2-chlorotrityl resin was added to a dry RV and initially swelled in 5 mL DMF for 30 min with magnetic stirring. Between each reaction, the resin was washed extensively with adequate DMF, DCM, and DMF. If the first amino acid was Fmoc-β-(7-methoxycoumarin-4-yl)-Ala-OH, 2 equiv. of the amino acid was dissolved in 1 mL of DMF with 4 equiv. of DIPEA and stirred for 30 min; this reaction was repeated to ensure efficient coupling. A methanol capping step with 5% methanol in DMF could be done after the first amino acid coupling to ensure that there was no reactive resin. After washing, the resin was incubated with 2 mL of 20% piperidine solution in DMF for 20 mins under stirring for deprotection. The same deprotection procedure was followed for all the subsequent standard amino acids. For a typical 45-minute coupling reaction, 5 equiv. of the standard amino acid and 5 equiv. of PyAOP was dissolved in 1 mL DMF, and added to the RV, with an addition of 10 equiv. of DIPEA. Thioamide residues were coupled and deprotected with slightly modified procedures. Thioamides were coupled through pre-activated precursors, where 3 equiv. of the thioamide precursor was dissolved in 1.5 mL of dry dichloromethane (DCM) with 5 equiv. DIPEA and stirred for 45 minutes. This procedure was repeated prior to deprotection to ensure efficient incorporation of the thioaminoacid onto the peptide chains. For the deprotection of thioamides, 2 mL of 2% DBU (1,8-diazabicyclo(5.4.0)undec-7-ene) in DMF was added to the RV and reacted three times for 2 min each, with extensive washing with DMF and DCM between each deprotection step.

Peptide Cleavage and Purification. Upon completion of the synthesis, the resin was dried with DCM under vacuum. Peptides were cleaved from resin by treatment with a 1-2 mL fresh cleavage cocktail of trifluoroacetic acid (TFA), water, and triisopropylsilane (TIPSH) (95:2.5:2.5 v/v) for 45 mins with stirring. After treatment, the cocktail solution was expelled from the RV with nitrogen and reduced to a volume of less than 1 mL by rotary evaporation. This resulting solution was then treated with over 10 mL of cold ethyl ether to precipitate out the peptides. This precipitate was flash frozen with liquid nitrogen and evaporated using lyophilization. The crude peptide was diluted in CH_3CN/H_2O (10:90 v/v) and then purified on a Luna® Omega 5 µm PS C18 100 Å, LC semi-preparative column (Phenomenex; Torrance, CA, USA) by HPLC using the following gradients at a flow rate of 3 mL/min; the full-length peptide was purified using a C4 column (**Table S1** and **Table S2**). The HPLC chromatograms of the first-pass purification are shown in **Figures S1– S10**. MALDI MS was used to confirm peptide identities; the identified masses summarized in **Table S3** and the MALDI MS spectra are shown in **Figures S1-S10**. Purified peptides were dried on a lyophilizer (Labconco; Kansas City, MO, USA) or in a vacuum centrifuge (Savant/Thermo Scientific; Rockford, IL, USA). If necessary, peptides were subjected to multiple rounds of purification until 99% purity by analytical HPLC was achieved.

Table S1. Peptide Purification Methods and Retention Time.

Peptide	Gradient	Retention Time
$NPY_{1\text{-}36}\text{-}Y^S_1$	1	25.3 min
$NPY_{1\text{-}36}\text{-}P^S_2$	1	25.4 min
YPSKPμ	2	17.4 min
Y^SPSKPμ	2	17.1 min
YPLKPμ	3	15.6 min
Y^SPLKP-μ	3	18.7 min
YPIKPμ	3	16.0 min
Y^SPIKP-μ	3	16.5 min
APLEP-μ	3	19.2 min
A^SPLEP-μ	3	19.5 min

* Abbreviations: μ: 7-methoxycoumarinylalanine; Y^S: thiotyrosine; P^S: thioproline; A^S: thioalanine.

Table S2. HPLC Gradients for Peptide Purification.

No.	Time (min)	% B	No.	Time (min)	% B
1	0:00	10	2	0:00	10
	2:00	20		3:00	10
	5:00	20		5:00	10
	35:00	50		25:00	30
	36:00	50		27:00	100
	37:00	100		30:00	100
	39:00	100		34:00	10
	43:00	10		35:00	10
	45:00	10			
3	0:00	10			
	3:00	20			
	5:00	20			
	25:00	40			
	27:00	100			
	30:00	100			
	34:00	10			
	35:00	10			

* Solvent A: 0.1 % TFA in water; Solvent B: 0.1 % TFA in acetonitrile

Table S3. Calculated and Observed Masses of Peptides.

Peptide	$[M+H]^+$		$[M+Na]^+$		$[M+K]^+$	
	Calculated	Observed	Calculated	Observed	Calculated	Observed
$NPY_{1\text{-}36}$-Y^S_1	4287.05	4287.145	4309.03	-	4325.00	-
$NPY_{1\text{-}36}$-P^S_2	4287.05	4287.729	4309.03	-	4325.00	-
YPSKPμ	836.39	836.054	858.37	858.067	874.34	874.035
Y^SPSKPμ	852.36	852.017	874.24	874.010	890.31	890.606
YPLKPμ	863.00	862.471	884.98	884.457	901.09	900.432
Y^SPLKPμ	879.06	878.449	901.04	900.429	917.15	916.404
YPIKPμ	862.44	862.626	884.320	884.605	900.39	900.172
Y^SPIKPμ	878.41	878.140	900.39	900.118	916.37	916.084
APLEPμ	771.85	771.415	793.83	793.398	809.94	809.371
A^SPLEPμ	787.91	787.394	809.89	809.378	826.00	825.382

* Abbreviations: μ: 7-methoxycoumarinylalanine; Y^S: thiotyrosine; P^S: thioproline; A^S: thioalanine

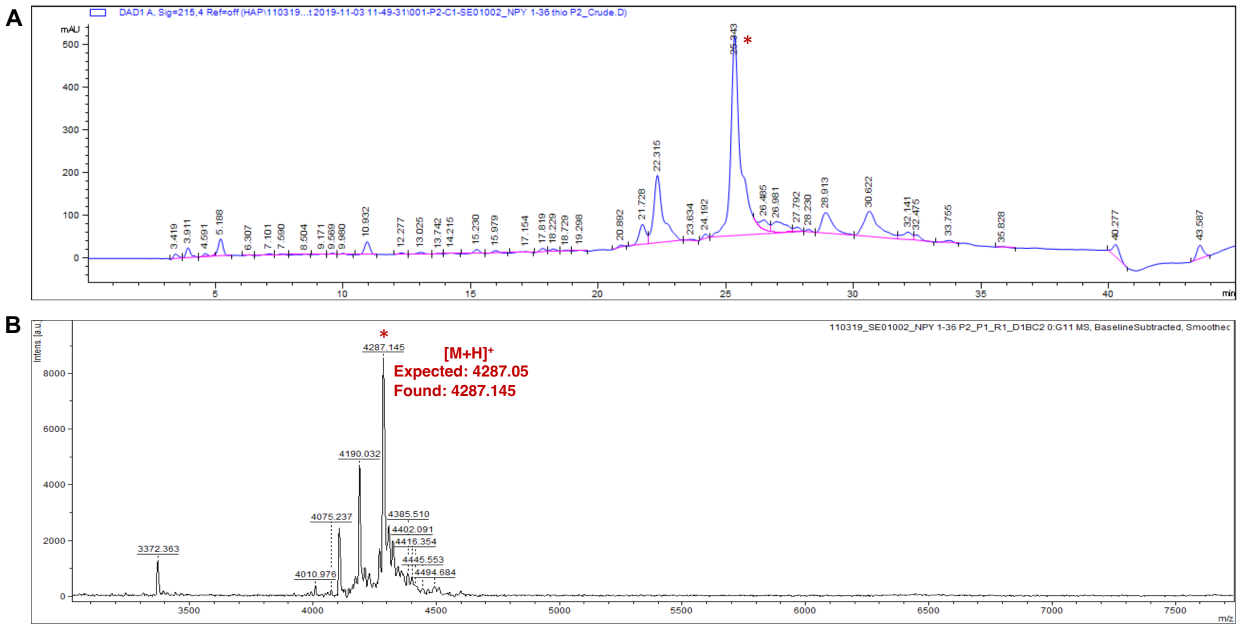

Figure S1. Purification and Characterization of NPY$_{1\text{-}36}$-Y$^{S}_{1}$ Peptide. (A) HPLC chromatogram of crude peptide sample. (B) MALDI-TOF MS spectrum of the peptide. The stars indicate the peak for the peptide product and its identified mass.

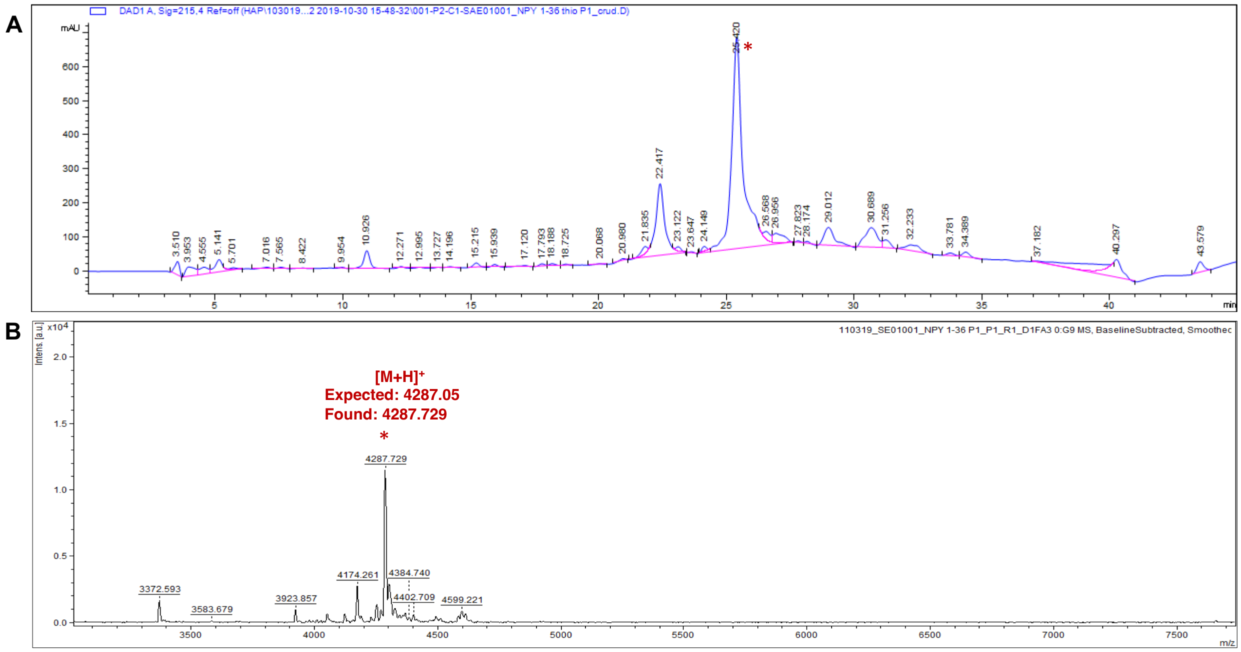

Figure S2. Purification and Characterization of NPY$_{1\text{-}36}$-P$^{S}_{2}$ Peptide. (A) HPLC chromatogram of crude peptide sample. (B) MALDI-TOF MS spectrum of the peptide. The stars indicate the peak for the peptide product and its identified mass.

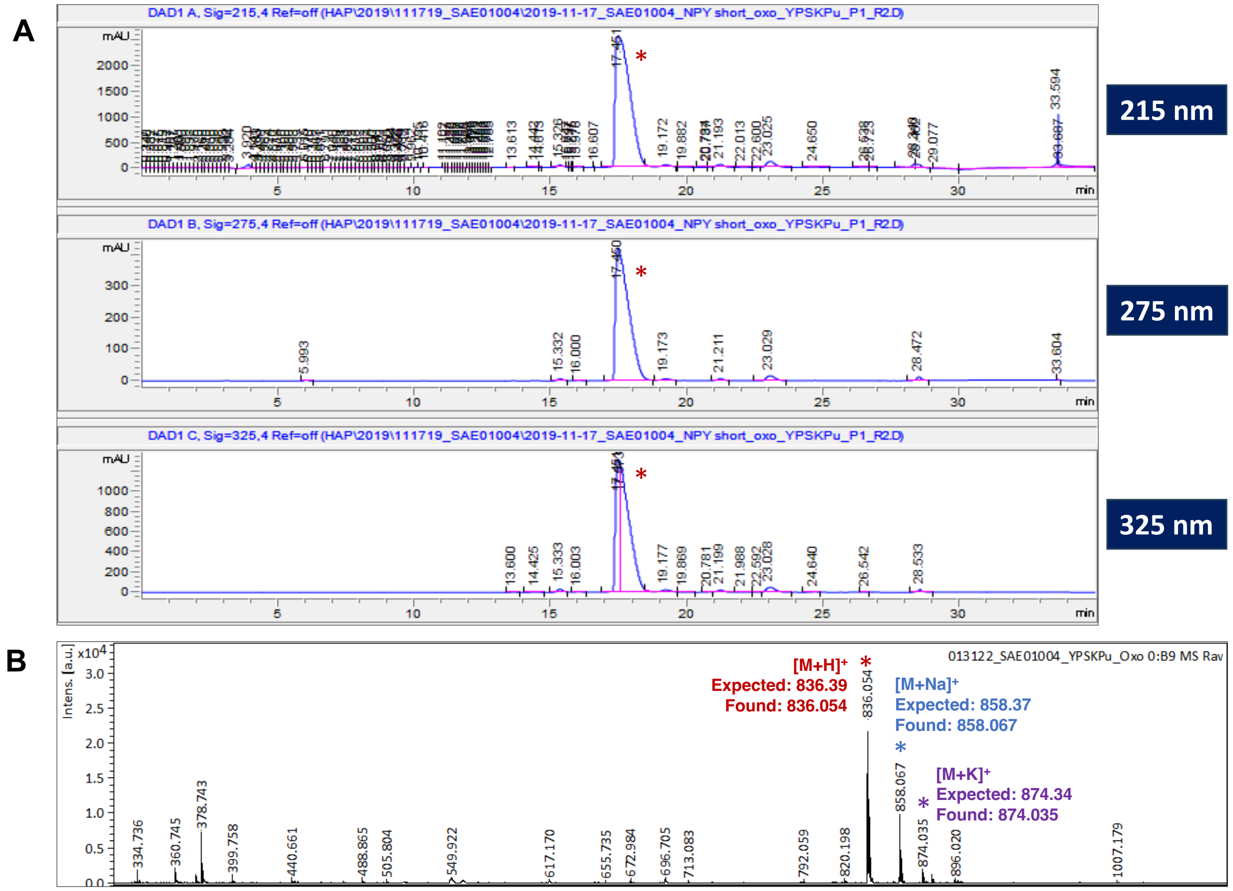

Figure S3. Purification and Characterization of YPSKPµ Peptide. (A) HPLC chromatogram of crude peptide sample; different wavelengths were monitored - 215 nm, 275 nm, and 325 nm. (B) MALDI-TOF MS spectrum of the peptide. The stars indicate the peak for the peptide product and its identified masses.

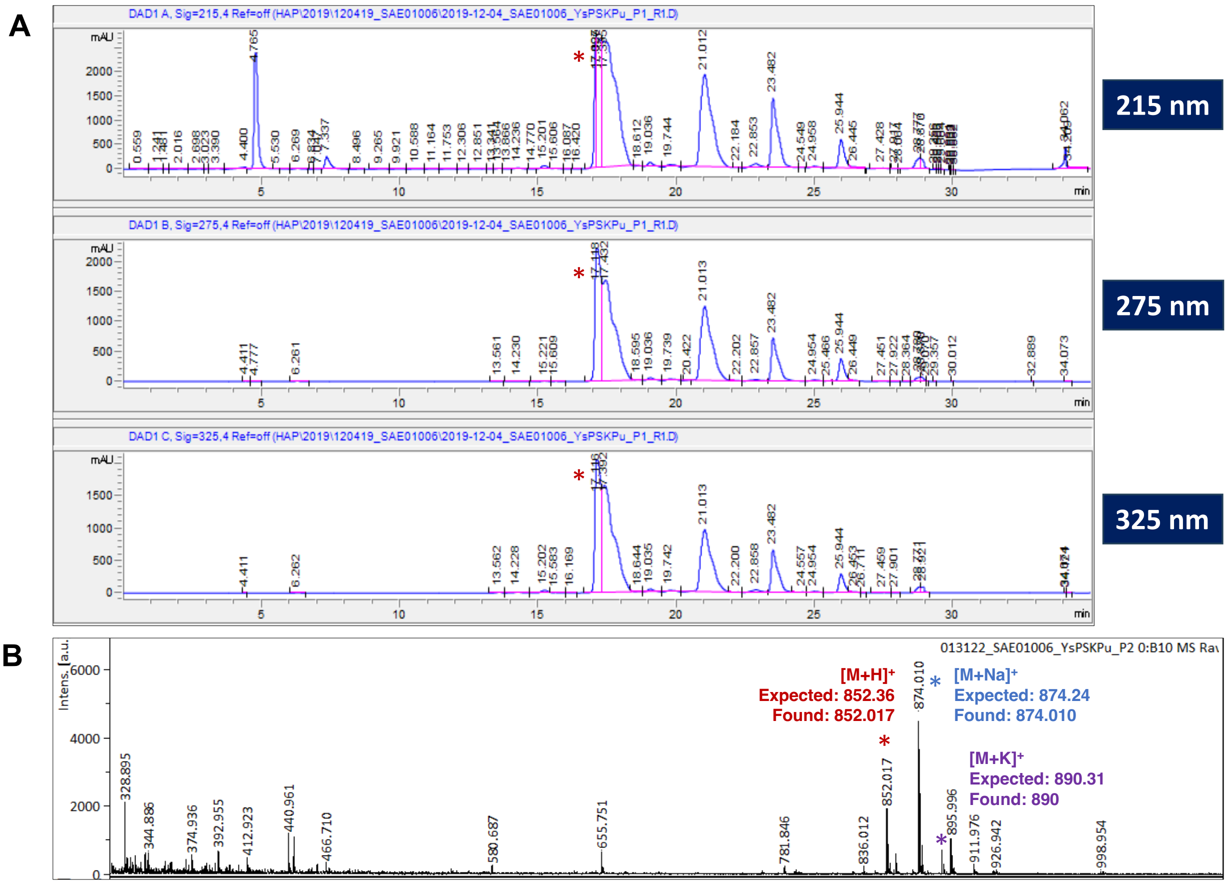

Figure S4. Purification and Characterization of Y^{S}PSKPμ Peptide. (A) HPLC chromatogram of crude peptide sample; different wavelengths were monitored - 215 nm, 275 nm, and 325 nm. (B) MALDI-TOF MS spectrum of the peptide. The stars indicate the peak for the peptide product and its identified masses.

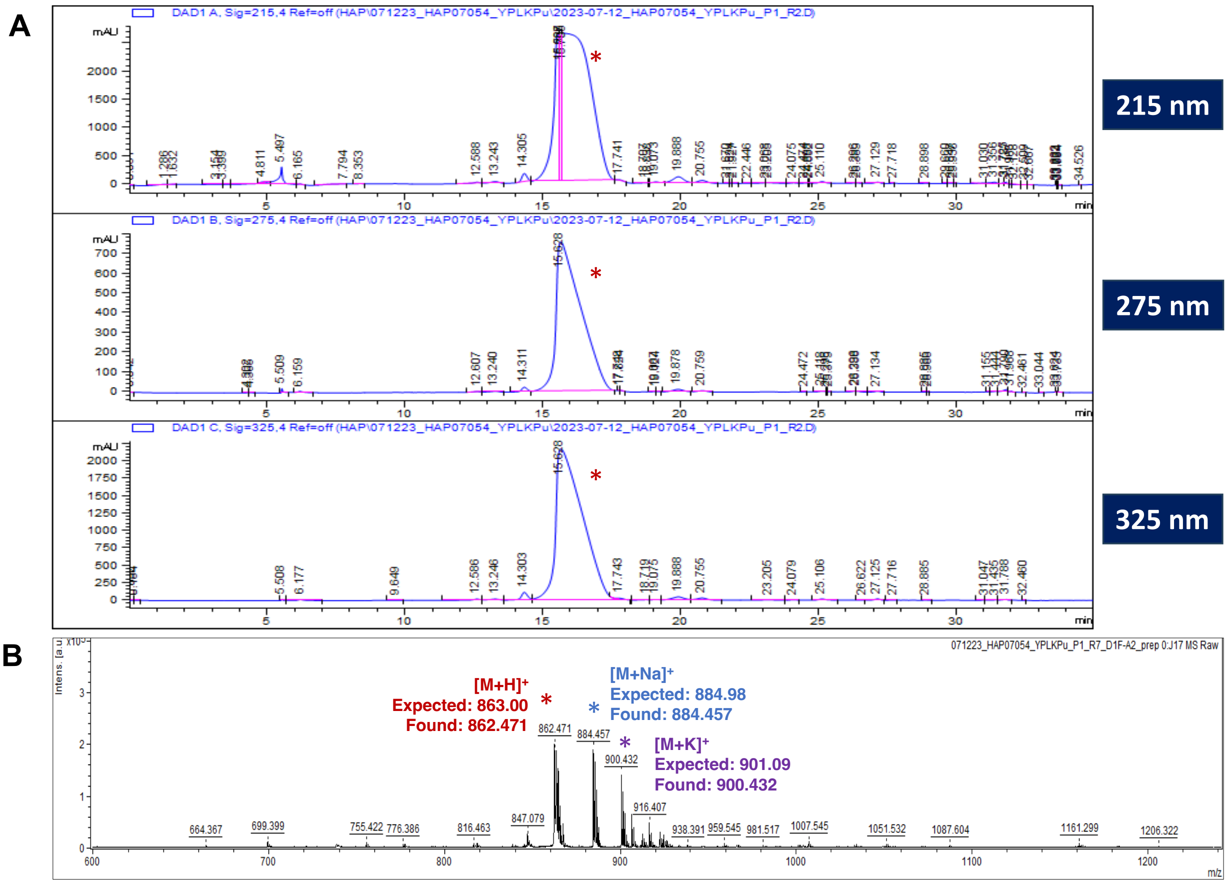

Figure S5. Purification and Characterization of YPLKPμ Peptide. (A) HPLC chromatogram of crude peptide sample; different wavelengths were monitored - 215 nm, 275 nm, and 325 nm. (B) MALDI-TOF MS spectrum of the peptide. The stars indicate the peak for the peptide product and its identified masses.

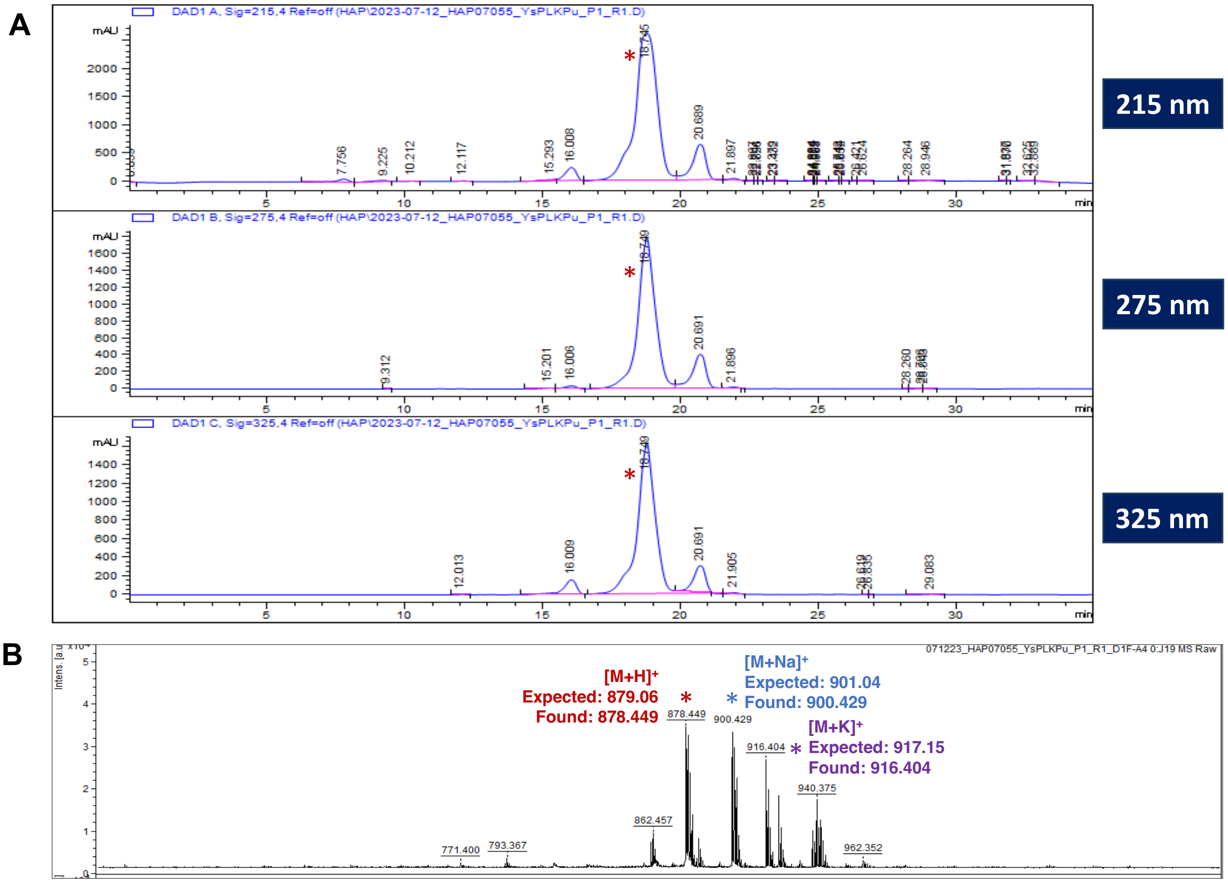

Figure S6. Purification and Characterization of Y^{S}PLKPμ Peptide. (A) HPLC chromatogram of crude peptide sample; different wavelengths were monitored - 215 nm, 275 nm, and 325 nm. (B) MALDI-TOF MS spectrum of the peptide. The stars indicate the peak for the peptide product and its identified masses.

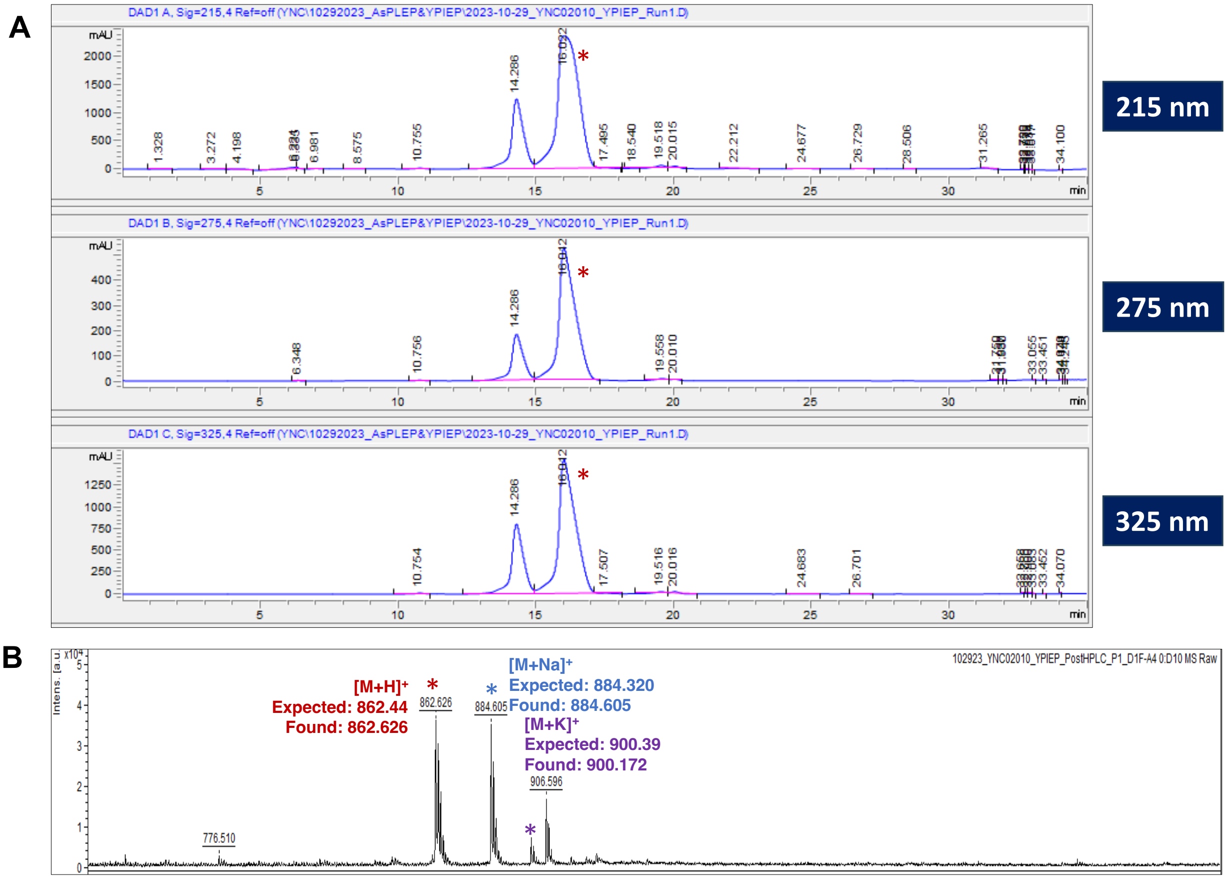

Figure S7. Purification and Characterization of YPIKPμ Peptide. (A) HPLC chromatogram of crude peptide sample; different wavelengths were monitored - 215 nm, 275 nm, and 325 nm. (B) MALDI-TOF MS spectrum of the peptide. The stars indicate the peak for the peptide product and its identified masses.

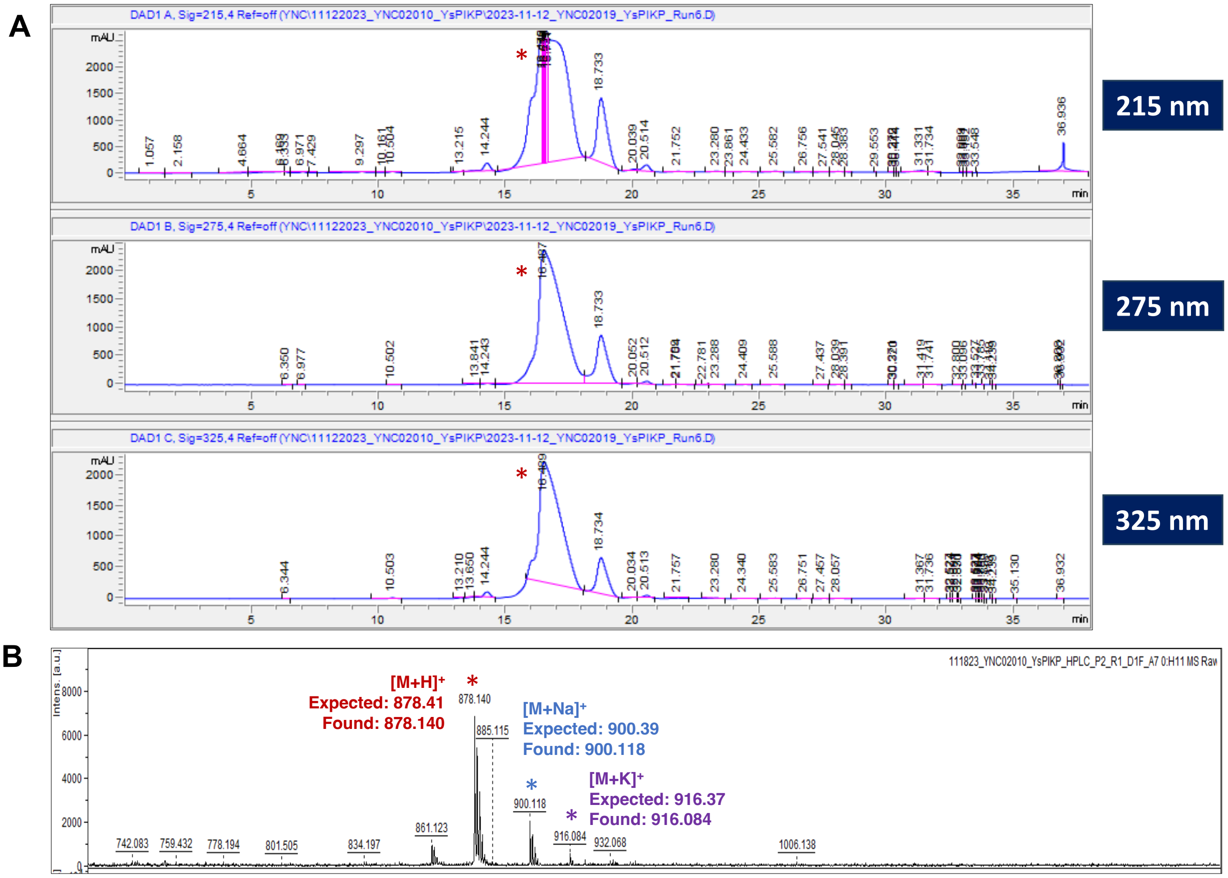

Figure S8. Purification and Characterization of Y^{S}PIKPµ Peptide. (A) HPLC chromatogram of crude peptide sample; different wavelengths were monitored - 215 nm, 275 nm, and 325 nm. (B) MALDI-TOF MS spectrum of the peptide. The stars indicate the peak for the peptide product and its identified masses.

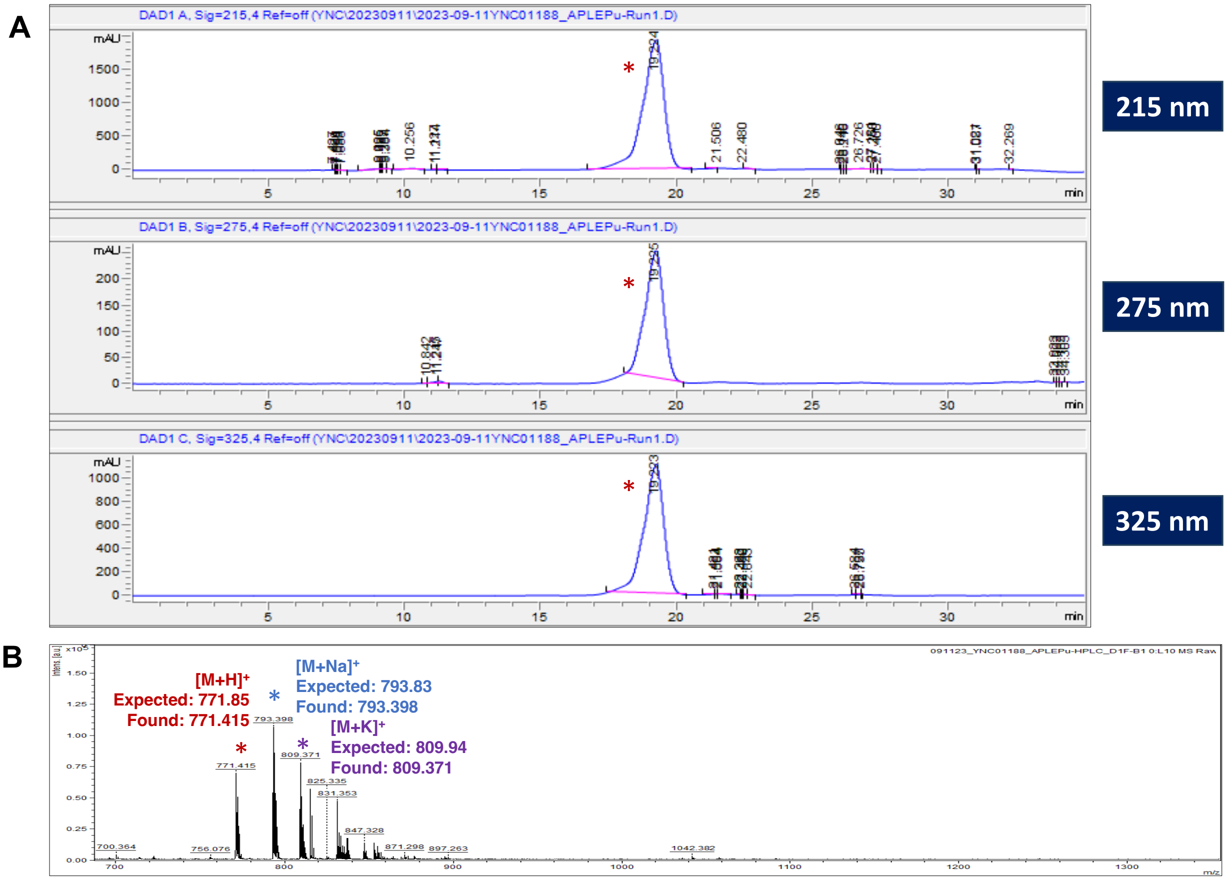

Figure S9. Purification and Characterization of APLEPµ Peptide. (A) HPLC chromatogram of crude peptide sample; different wavelengths were monitored - 215 nm, 275 nm, and 325 nm. (B) MALDI-TOF MS spectrum of the peptide. The stars indicate the peak for the peptide product and its identified masses.

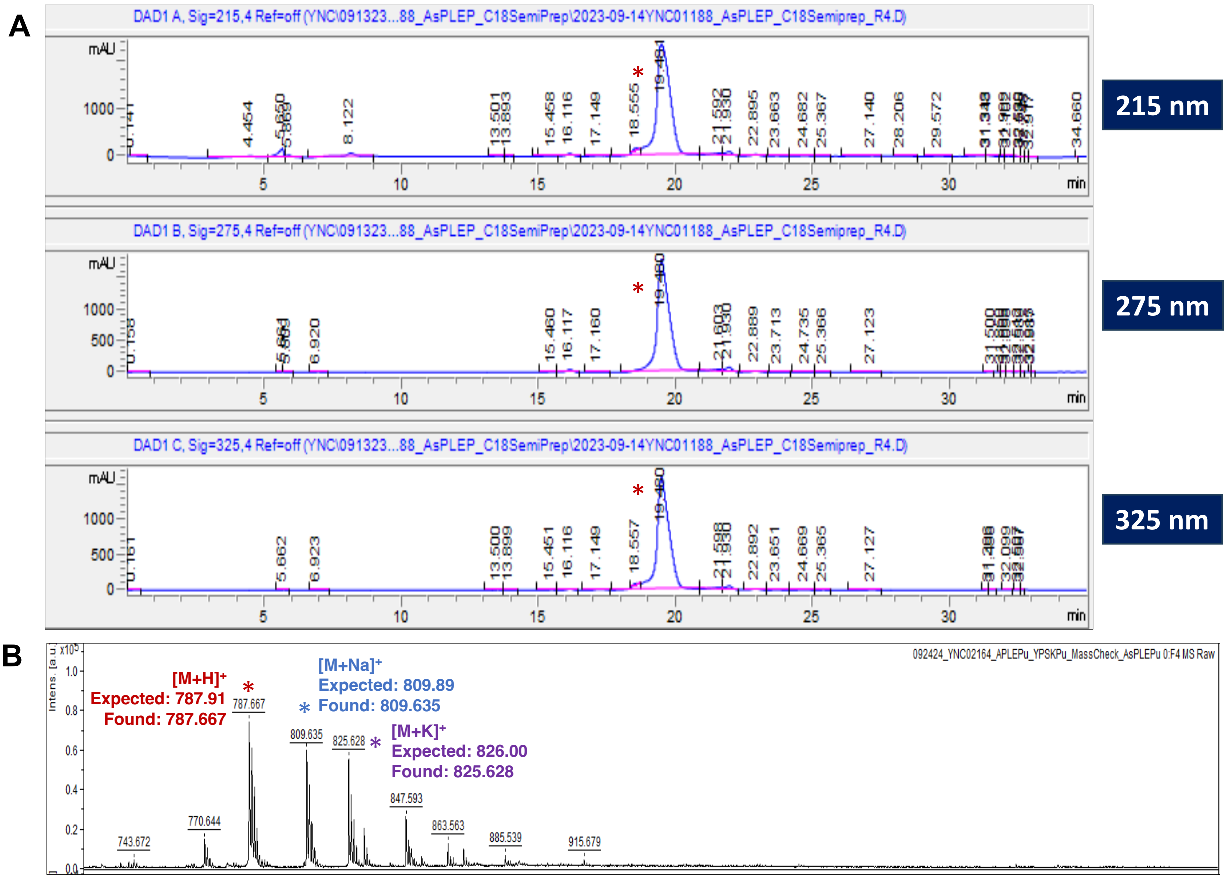

Figure S10. Purification and Characterization of A^{S}PLEPμ Peptide. (A) HPLC chromatogram of crude peptide sample; different wavelengths were monitored - 215 nm, 275 nm, and 325 nm. (B) MALDI-TOF MS spectrum of the peptide. The stars indicate the peak for the peptide product and its identified masses.

3. DPP-4 Proteolysis Assays with Full-Length NPY Peptides (HPLC)

A 19 µL solution of 105.3 µM peptide in DPBS and 26.3 µM Trp internal standard was incubated at 37 °C in the presence of 1 µL of 25 ng/µL DPP-4 (Sigma Aldrich, D3446), to a final concentration of 100 µM peptide, 25 µM Trp, and 1.25 ng/µL DPP-4. After incubating for the desired time (for all-amide t = 0, 2, 5, 8, and 12 minutes), the reaction was then quenched with 2 µL of 1 M hydrochloric acid (HCl). For the thioamide peptides, assays were done with longer incubation time: $NPY_{1\text{-}36}$-Y^S_1 (t = 0, 5, 10, 20, and 30 minutes) and $NPY_{1\text{-}36}$-P^S_2 (t = 0, 1, 4, and 24 hours). To prepare samples for analysis with analytical HPLC, the reaction was diluted to 200 µL with Milli-Q. Samples were run in triplicate for each time point. All samples were analyzed by an Agilent 1260 Infinity II series Analytical HPLC using a Phenomenex Luna C8(2) Analytical column (Torrance, CA, USA) (**Figures S11 – S13**). All peptides were monitored at 280 nm and the amount of intact peptide was quantified by integrating peak areas. To determine the percent intact peptide in each sample, the internal standard was used for normalization of the amount of intact peptide; the average ratio of intact peptide from the three trials to internal standard was then compared to the ratio at t = 0 minutes. MALDI-MS was used to confirm the identity of the intact peptide and its cleavage products (**Figures S11 – S13**). A Phenomenex Luna® Omega 5 µm PS C18 100 Å analytical HPLC column was used to analyze all samples (Solvent A: 0.1% TFA in Milli-Q water; Solvent B: 0.1% TFA in acetonitrile).

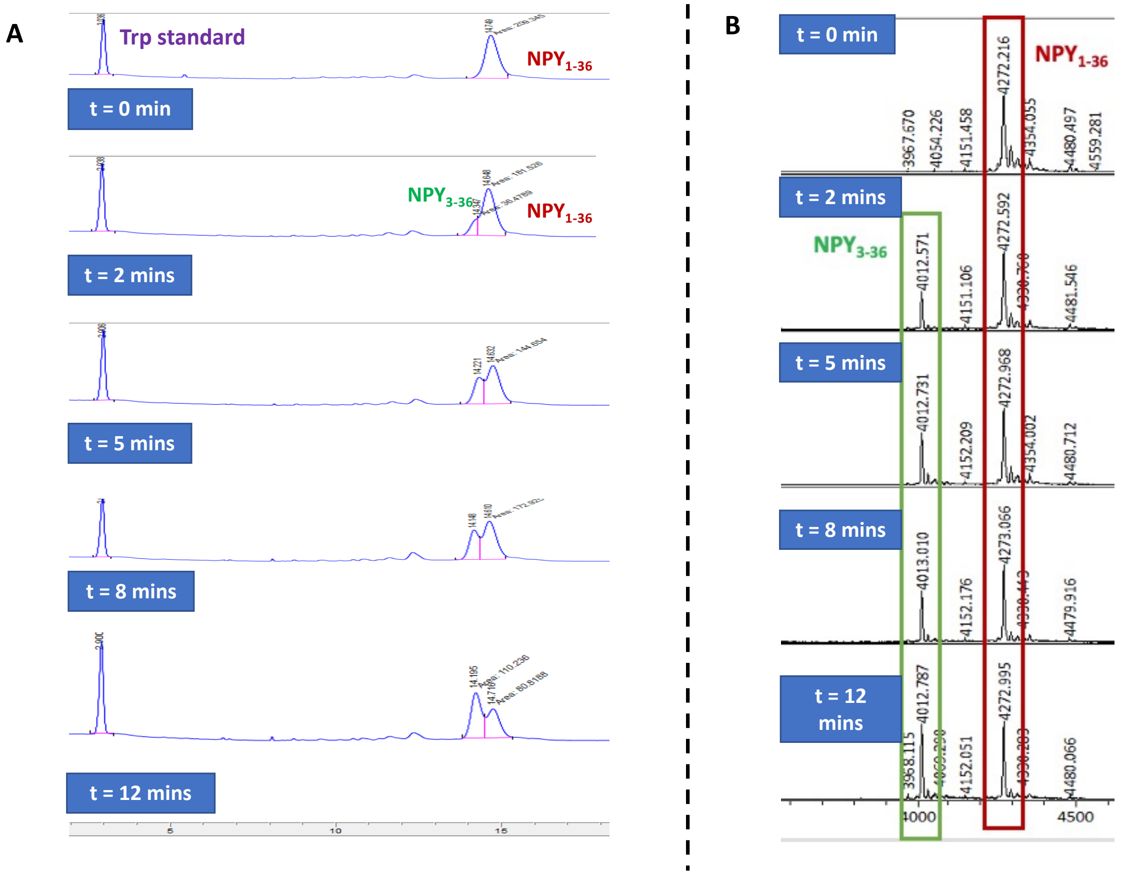

Figure S11. DPP-4 Proteolysis Assay with all-amide, full-length $NPY_{1\text{-}36}$. (A) Analytical HPLC traces and (B) MALDI at different time points. Expected $[M+H]^+$ for $NPY_{1\text{-}36}$ = 4271.08 (found masses indicated with the red box); Expected $[M+H]^+$ for $NPY_{3\text{-}36}$ = 4010.96 (found masses indicated with the green box).

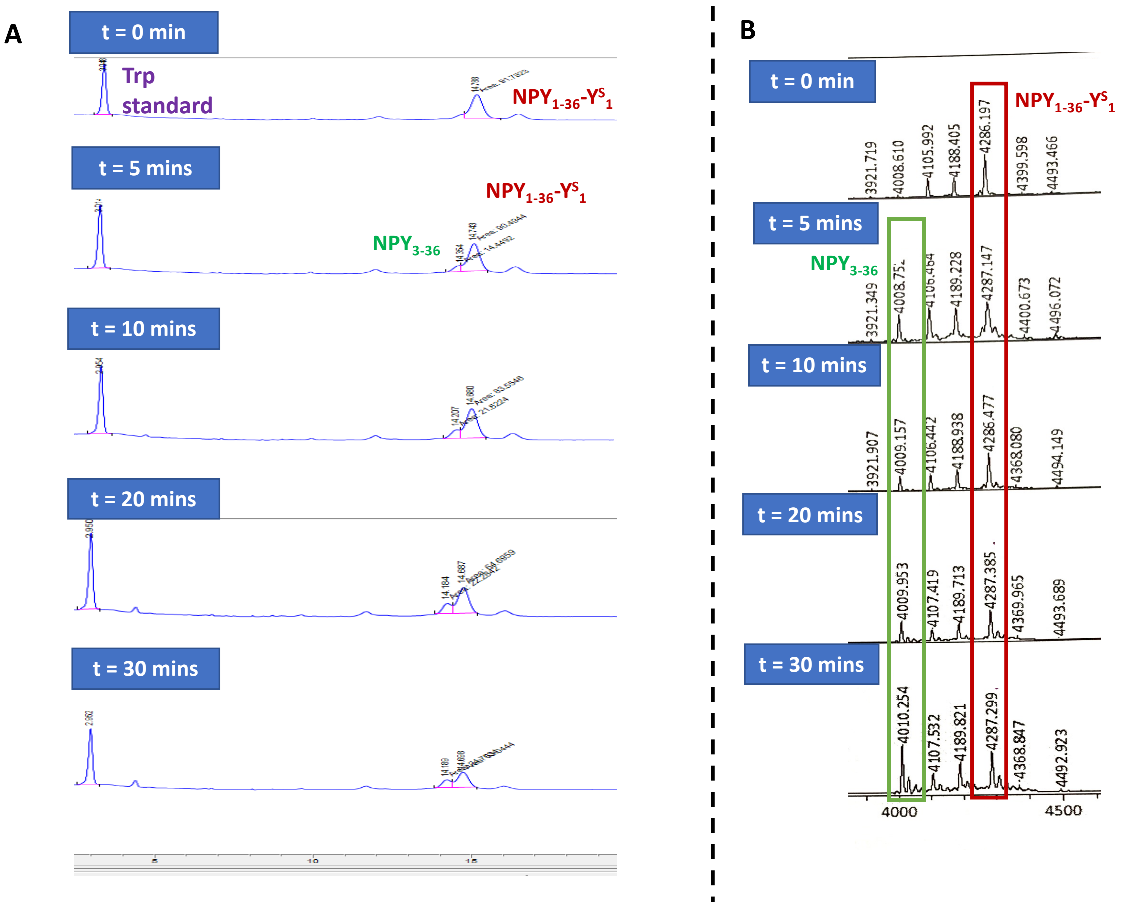

Figure S12. DPP-4 Proteolysis Assay with P2 thioamide, full-length $NPY_{1\text{-}36}\text{-}Y^{S}_{1}$. (A) Analytical HPLC traces and (B) MALDI at different time points. Expected $[M+H]^+$ for $NPY_{1\text{-}36}$ = 4287.05 (found masses indicated with the red box); Expected $[M+H]^+$ for $NPY_{3\text{-}36}$ = 4010.96 (found masses indicated with red box).

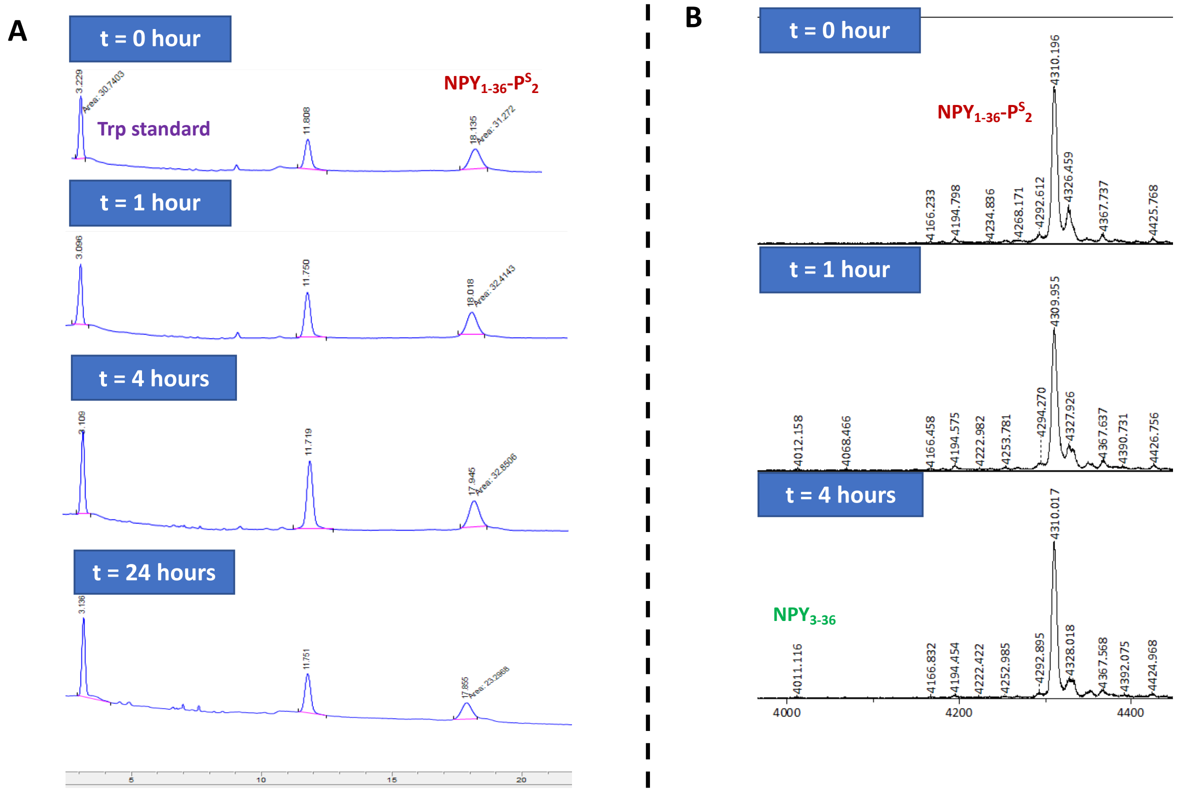

Figure S13. DPP-4 Proteolysis Assay with P1 thioamide, full-length NPY$_{1\text{-}36}$-P$^{S}_{2}$. (A) Analytical HPLC traces and (B) MALDI at different time points. Expected $[M+H]^+$ for NPY$_{1\text{-}36}$ = 4287.05; Expected $[M+H]^+$ for NPY$_{3\text{-}36}$ = 4010.96.

4. DPP-4 Proteolysis Assays (Steady State Assays)

A 19 μL solution of 5.3 μM peptide was incubated in the absence or presence of 1 μL of 46.2 ng/μL DPP-4 (Sigma Aldrich, D3446), to final concentrations of 5 μM peptide and 2.31 ng/μL DPP-4 in DPBS buffer and pH 7.6 at 27°C. For the PP-based probes (APLEPμ & A^{S}PLEPμ), additional assays were conducted at higher DPP-4 concentration of 4.62 ng/μL to improve the cleavage rate and kinetics of the thioamide probes (**Figure S17B**). For the μ-tagged probes, the fluorescence was monitored as a function of time at 390 nm with an excitation wavelength of 325 nm on the Tecan M1000 plate reader. Three replicates were performed for each assay to ensure reproducibility. These primary data are shown. At the end of the plate-reader assay, the sample was diluted to a final volume of 170 μL for validation with HPLC. MALDI MS & HPLC analysis of peptide proteolysis by DPP-4 were also done to confirm the cleavage sites, indicated by the slashes (*e.g.* YP/SKPμ) as summarized in **Figures S14-S17**, and **Tables S4-S7**. A Phenomenex Luna® Omega 5 μm PS C18 100 Å analytical HPLC column was used to analyze all samples using the same gradient of 20-25 % B over 35 minutes (Solvent A: 0.1% TFA in Milli-Q water; Solvent B: 0.1% TFA in acetonitrile).

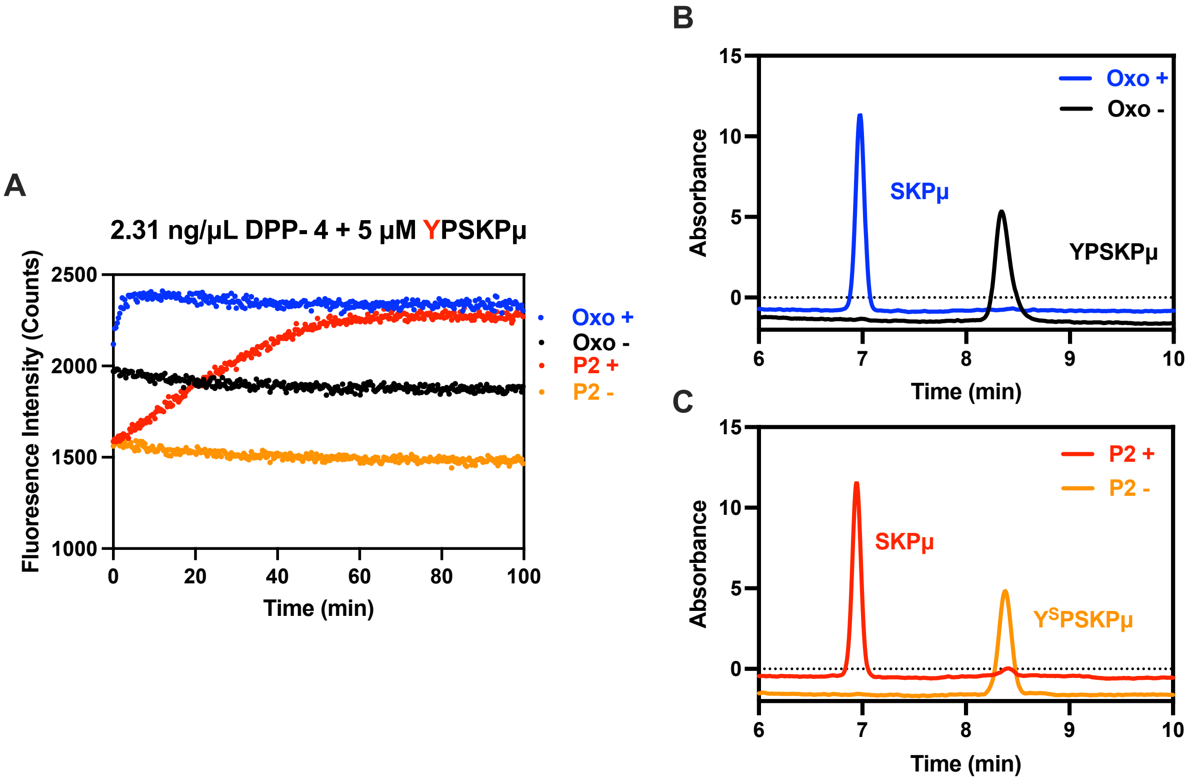

Figure S14. DPP-4 Proteolysis of NPY-based Probes (YPSKPμ and Y^{S}PSKPμ). (A) Raw fluorescence traces. Fluorescence turn-on by both an all-amide (in the presence of the intrinsic Y-μ quenching) and a P2 thioamide sensor can be used to monitor DPP-4 cleavage. The (-) indicates in the absence of the protease and the (+) indicates the presence of the protease. The fluorescence was monitored as a function of time at 390 nm with an excitation wavelength of 325 nm. All traces show the average of three replicates. (B) & (C) HPLC analysis of NPY-based probes cleaved by DPP-4. Reaction mixtures from fluorescence assays were analyzed by HPLC at 325 nm after completion of the reactions at the end of the assays.

Table S4. Masses Identified from DPP-4 Protease Assays with YPSKPμ and Y^{S}PSKPμ probes.

Condition	Fragment	[M+H]$^{+}$		[M+Na]$^{+}$		[M+K]$^{+}$	
		Calculated	Observed	Calculated	Observed	Calculated	Observed
**No protease** ***(Negative Control)***	YPSKPμ *(Intact peptide)*	836.39	836.224	858.37	858.207	874.34	874.180
**+ DPP-4**	SKPμ	576.26	576.158	598.24	598.123	614.21	-
**No protease**	Y^{S}PSKPμ *(Intact peptide)*	852.36	852.203	874.24	874.178	890.31	890.158
**+ DPP-4**	SKPμ	576.26	576.162	598.24	-	614.21	-

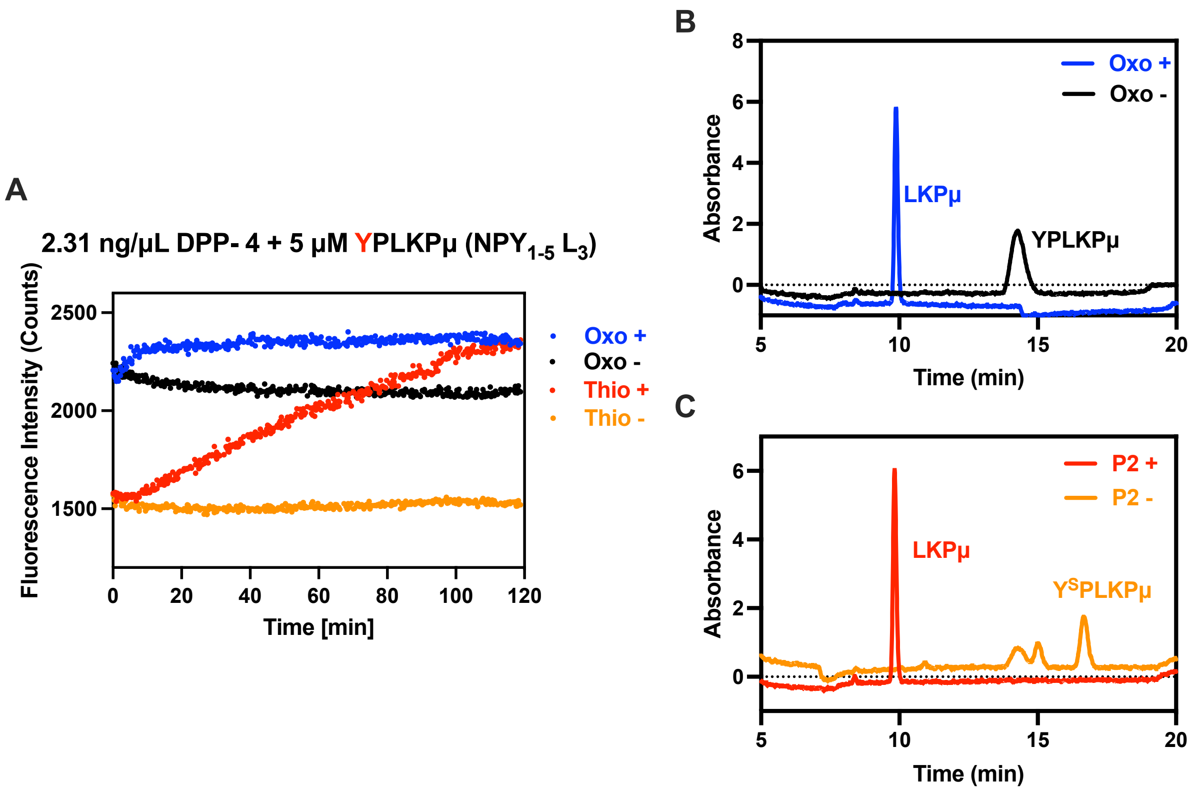

Figure S15. DPP-4 Proteolysis of P1' mutated NPY-based probes (YPLKPμ and Y^{S}PLKPμ). (A) Raw fluorescence traces. Fluorescence turn-on by both an all-amide (in the presence of the intrinsic Y-μ quenching) and a P2 thioamide sensor can be used to monitor DPP-4 cleavage. The (-) indicates in the absence of the protease and the (+) indicates the presence of the protease. The fluorescence was monitored as a function of time at 390 nm with an excitation wavelength of 325 nm. All traces show the average of three replicates. (B) & (C) HPLC analysis of P1' mutated NPY-based probes cleaved by DPP-4. Reaction mixtures from fluorescence assays were analyzed by HPLC at 325 nm after completion of the reactions at the end of the assays.

Table S5. Masses Identified from DPP-4 Protease Assays with YPLKPμ and Y^{S}PLKPμ probes.

**Condition**	**Fragment**	**[M+H]$^+$**		**[M+Na]$^+$**		**[M+K]$^+$**	
		**Calculated**	**Observed**	**Calculated**	**Observed**	**Calculated**	**Observed**
**No protease** ***(Negative Control)***	YPLKPμ *(Intact peptide)*	863.00	862.542	884.98	884.488	901.09	900.785
**+ DPP-4**	LKPμ	602.71	602.352	624.69	624.484	640.80	640.518
**No protease**	Y^{S}PLKPμ *(Intact peptide)*	879.06	878.728	901.04	900.749	917.15	916.382
**+ DPP-4**	LKPμ	602.71	602.471	624.69	624.407	640.80	-

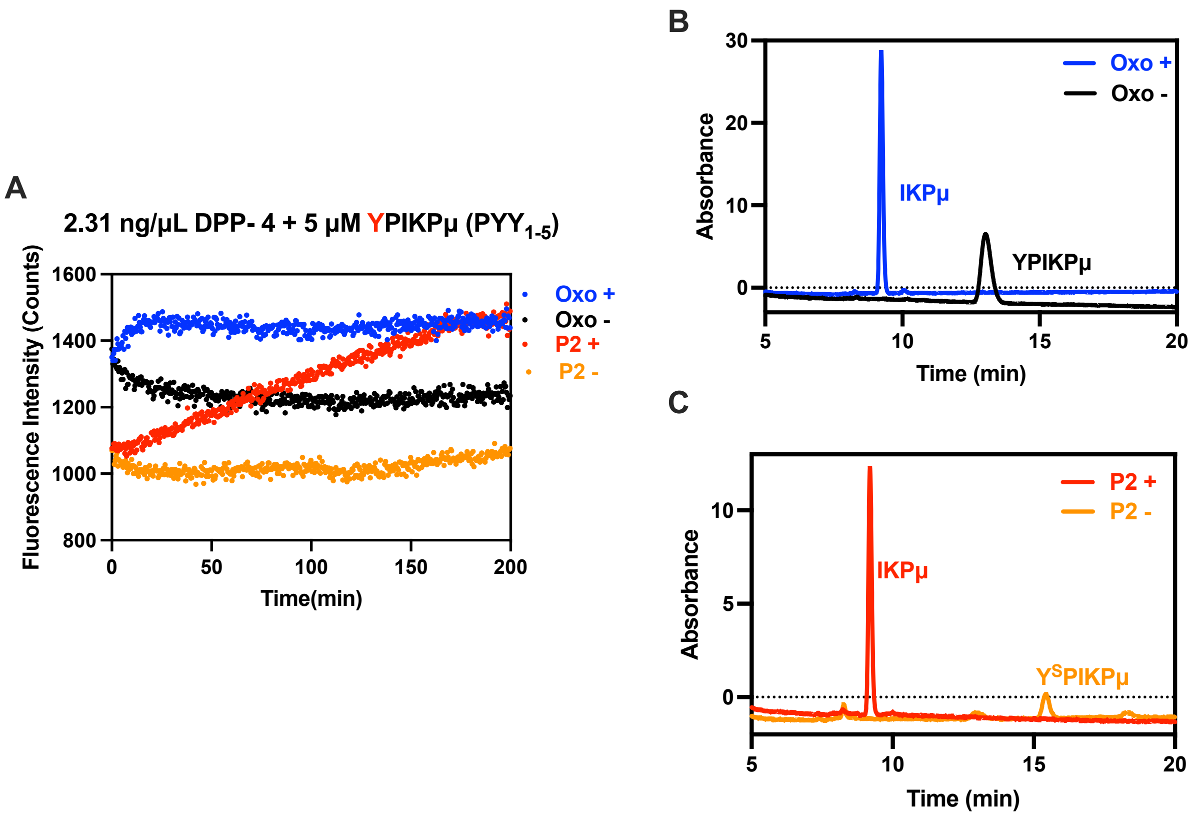

Figure S16. DPP-4 Proteolysis of PYY-based probes (YPIKPμ and Y^SPIKPμ). (A) Raw fluorescence traces. Fluorescence turn-on by both an all-amide (in the presence of the intrinsic Y-μ quenching) and a P2 thioamide sensor can be used to monitor DPP-4 cleavage. The (-) indicates in the absence of the protease and the (+) indicates the presence of the protease. The fluorescence was monitored as a function of time at 390 nm with an excitation wavelength of 325 nm. All traces show the average of three replicates. (B) & (C) HPLC analysis of PYY-based probes cleaved by DPP-4. Reaction mixtures from fluorescence assays were analyzed by HPLC at 325 nm after completion of the reactions at the end of the assays.

Table S6. Masses Identified from DPP-4 Protease Assays with YPIKPμ and Y^SPIKPμ probes.

Condition	Fragment	$[M+H]^+$		$[M+Na]^+$		$[M+K]^+$	
		**Calculated**	**Observed**	**Calculated**	**Observed**	**Calculated**	**Observed**
**No protease** ***(Negative Control)***	YPIKPμ *(Intact peptide)*	862.44	862.124	884.42	884.320	900.39	900.088
**+ DPP-4**	IKPμ	602.32	602.039	625.70	625.528	641.28	-
**No protease**	Y^SPIKPμ *(Intact peptide)*	878.41	878.221	900.39	900.139	916.37	916.159
**+ DPP-4**	IKPμ	602.32	602.146	625.70	625.269	641.28	-

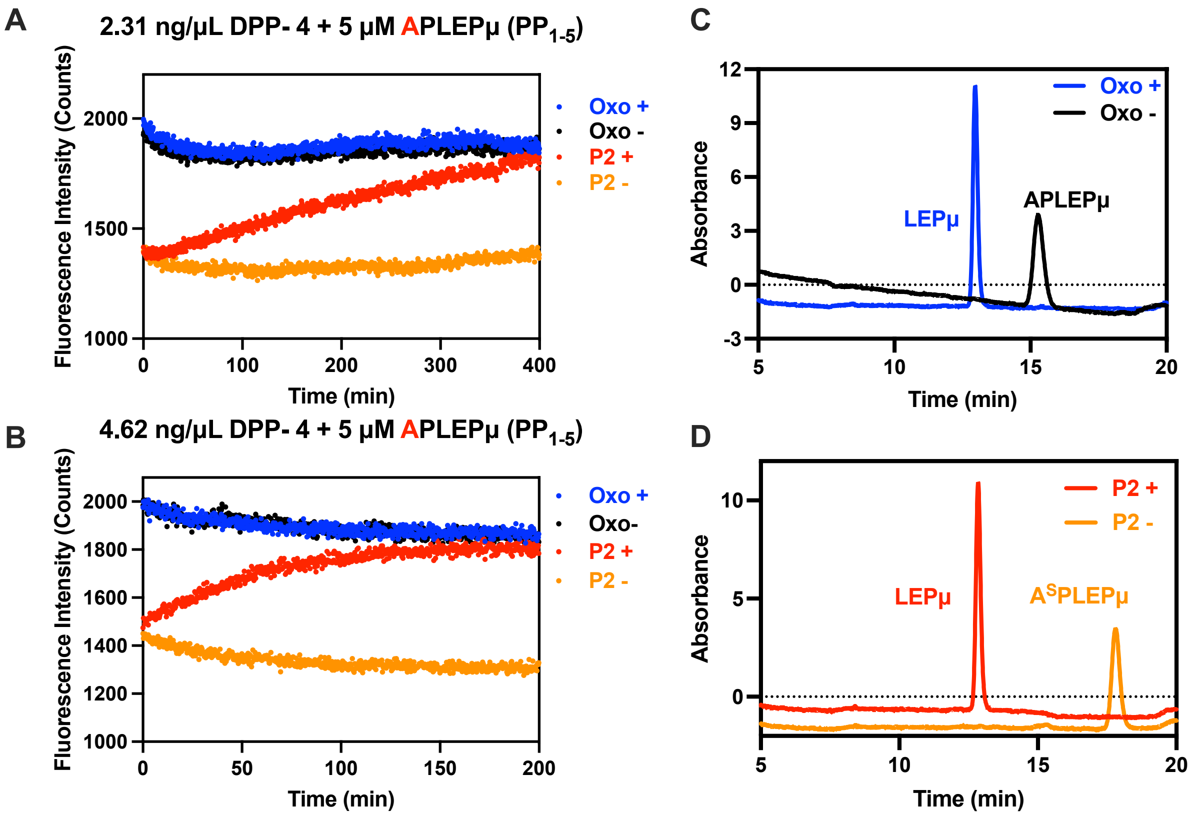

Figure S17. DPP-4 Proteolysis of PP-based probes (APLEPμ and A^{S}PLEPμ). (A) & (B) Raw fluorescence traces at 2.31 ng/μL DPP-4 and 4.62 ng/μL DPP-4. Fluorescence turn-on by both an all-amide (in the presence of the intrinsic Y-μ quenching) and a P2 thioamide sensor can be used to monitor DPP-4 cleavage. The (-) indicates in the absence of the protease and the (+) indicates the presence of the protease. The fluorescence was monitored as a function of time at 390 nm with an excitation wavelength of 325 nm. All traces show the average of three replicates. (C) & (D) HPLC analysis of PP-based probes cleaved by DPP-4. Reaction mixtures from fluorescence assays were analyzed by HPLC at 325 nm after completion of the reactions at the end of the assays.

Table S7. Masses Identified from DPP-4 Protease Assays with APLEPμ and A^{S}PLEPμ probes.

Condition	Fragment	$[M+H]^+$		$[M+Na]^+$		$[M+K]^+$	
		**Calculated**	**Observed**	**Calculated**	**Observed**	**Calculated**	**Observed**
**No protease *(Negative Control)***	APLEPμ *(Intact peptide)*	771.85	770.354	793.83	793.404	809.94	809.385
**+ DPP-4**	LEPμ	603.27	603.330	625.63	625.320	641.74	641.296
**No protease**	A^{S}PLEPμ *(Intact peptide)*	787.91	786.332	809.89	809.389	826.00	825.247
**+ DPP-4**	LEPμ	603.27	602.264	625.63	625.323	641.74	641.358

5. Sitagliptin Inhibition of DPP-4

The same protocol as the above DPP-4 proteolysis assays was used, except that the DPP-4 protease was pre-incubated either with or without sitagliptin (DPP-4 specific inhibitor, ≥98% by HPLC; Sigma-Aldrich SML3205) for 10 minutes before being added to the well containing the probe for the reactions. The data for the all-amide version of the PP-based probe (APLEPμ) was not included since there was no change in fluorescence intensity in the presence and the absence of the protease, as established in the previous assay (**Figure S17**). Sitagliptin is a DPP-4 inhibitor and has IC_{50} value of 18 nM. The final concentration of the inhibitor in the assays were 50 nM. That data for all the probes were shown in **Figure S19**. We also conducted the inhibition assays for the thioamide probes at the higher concentration of the inhibitor of 500 nM (**Figure S20**). We also did a test assay with the commercial DPP-4 probe GP-AMC (H-Gly-Pro-AMC; Anaspec AS-24098), in the absence and presence of sitagliptin, at the same final concentrations of 5 μM probe peptide, 2.31 ng/μL DPP-4, and with or without 50 nM sitagliptin in DPBS buffer and pH 7.6 at 27 °C. The assay with the commercial probe GP-AMC was monitored on the plate reader at the excitation wavelength of 380 nm and emission wavelength of 460 nm (**Figure S18**).

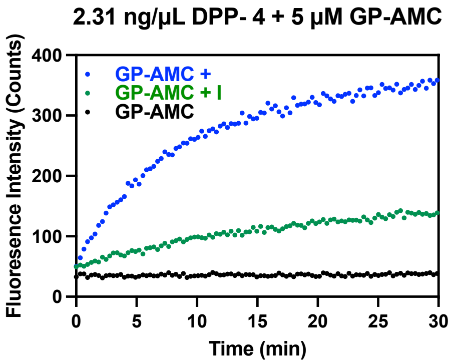

Figure S18. Commercial probe GP-AMC Cleaved by DPP-4 in the Presence of Absence of the Inhibitor Sitagliptin. The assays were done with the protease (+), with the protease & 50 nM of the inhibitor (+ I), and without both the protease and inhibitor (negative control; -). Fluorescence intensity was measured at 460 nm. All traces show the average of three replicates.

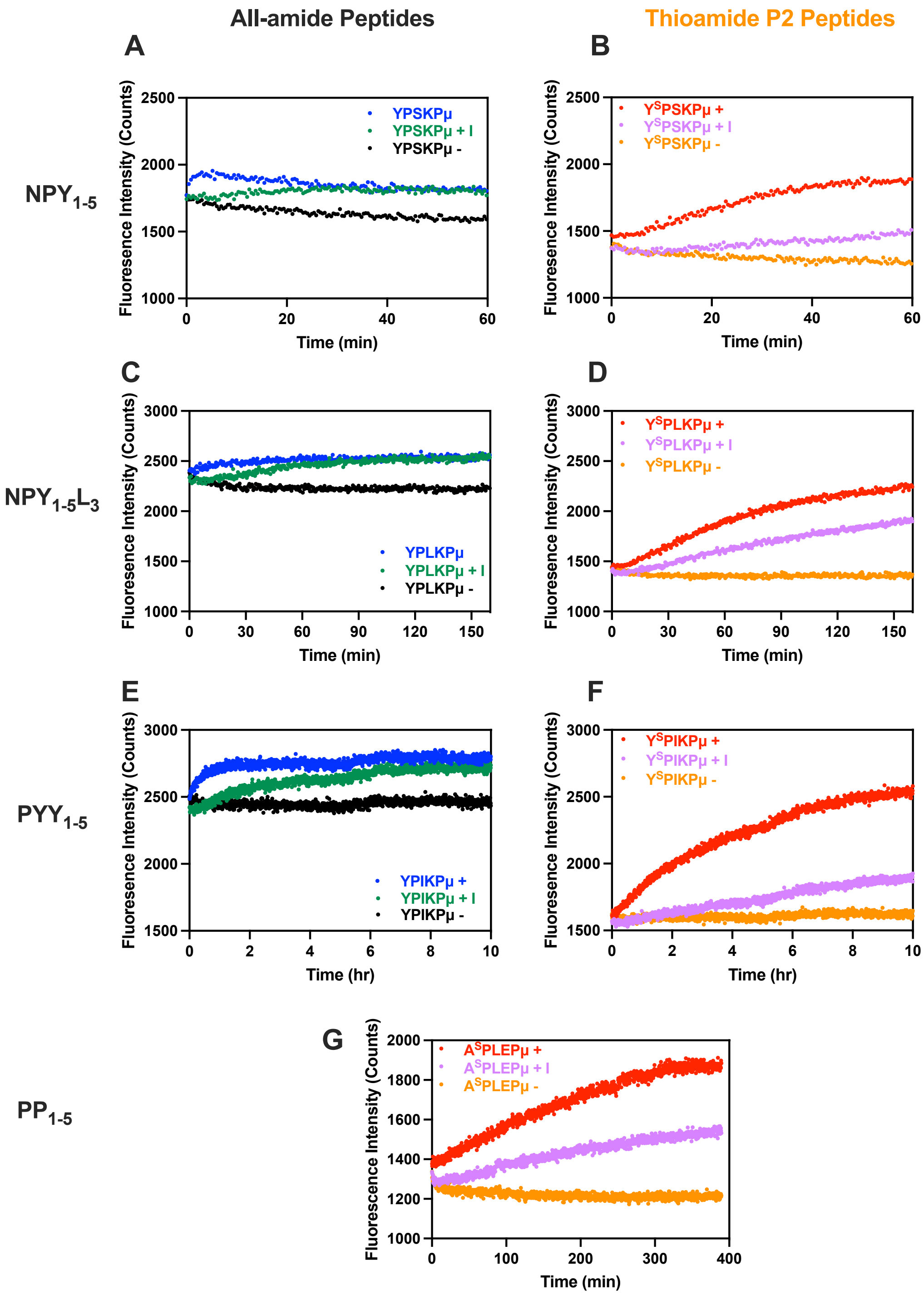

Figure S19. DPP-4 Inhibition by Sitagliptin Detected by Our Probes. For each protease, the assays were done with the protease (+), with the protease & 50 nM of the inhibitor (+ I), and without both the protease and inhibitor (negative control; -). Fluorescence intensity was measured at 325 nm. The fluorescence traces are shown for the probes: (A) & (B) NPY-based Probes (YPSKPμ & Y^SPSKPμ); (C) & (D) NPY-based Probes - P1'mutated (YPLKPμ & Y^SPLKPμ); (E) & (F) PYY-based probes (YPIKPμ & Y^SPIKPμ); (G) PP-based probe (A^SPLEPμ). All probes showed inhibition by sitagliptin, a specific inhibitor of DPP-4. All traces show the average of three replicates.

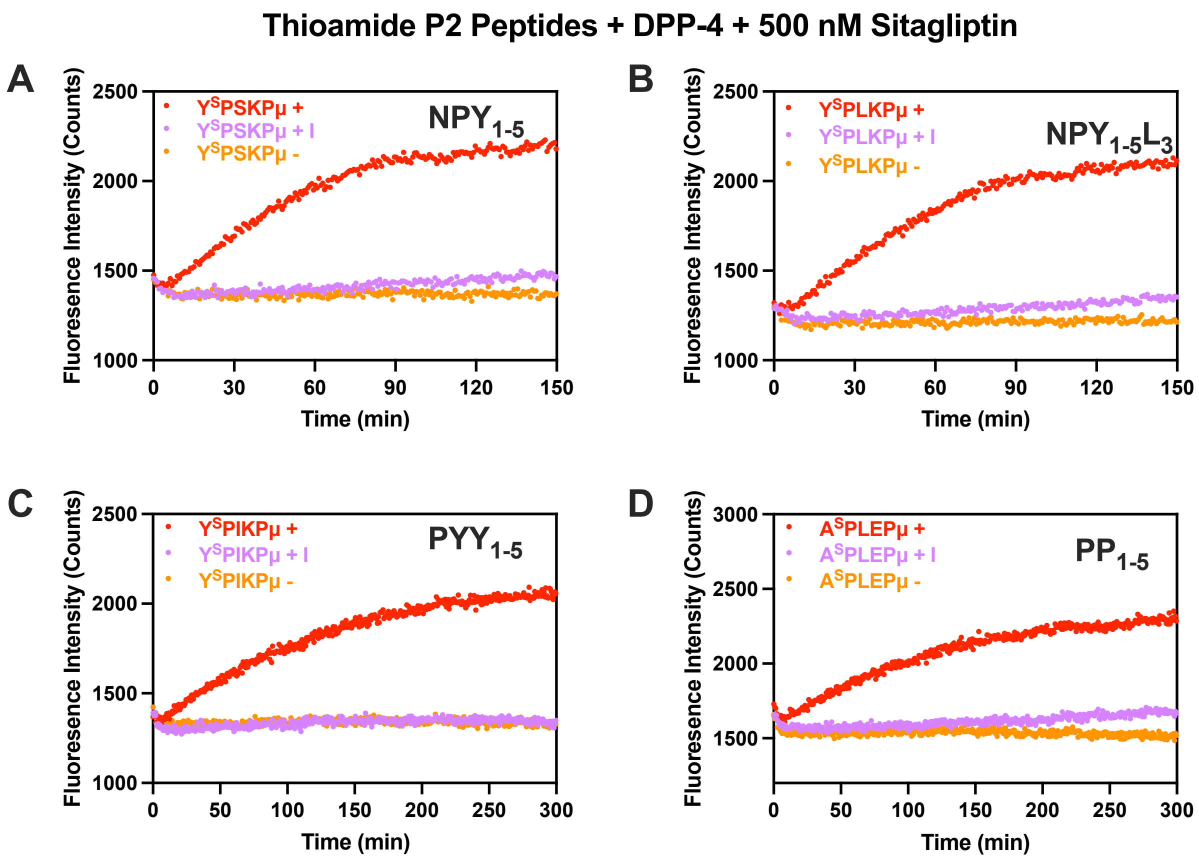

Figure S20. DPP-4 Inhibition by Sitagliptin Detected by Our Probes. For each protease, the assays were done with the protease (+), with the protease & 500 nM of the inhibitor (+ I), and without both the protease and inhibitor (negative control; -). Fluorescence intensity was measured at 325 nm. The fluorescence traces are shown for the probes: (A) NPY-based Thioamide Probe (Y^{S}PSKPμ); (B) NPY-based Thioamide Probe - P1'mutated (Y^{S}PLKPμ); (C) PYY-based Thioamide probe (Y^{S}PIKPμ); (G) PP-based probe (A^{S}PLEPμ). All thioamide probes showed inhibition by 500 nM sitagliptin and more inhibition compared to 50 nM sitagliptin as shown in Figure S19. All traces show the average of three replicates.

6. Human Saliva Assays

All assays were conducted with pooled human saliva commercially purchased from Innovative Research (Catalog #IRHUSL5ML). For each probe, 25 µL of human saliva was added to 25 µL of 10 µM probe to a final probe concentration of 5 µM. The probes were diluted in DPBS pH 7.6, and the assays were conducted at 27°C. For the µ-tagged probes, the fluorescence was monitored as a function of time at 390 nm with an excitation wavelength of 325 nm on the Tecan M1000 plate reader. Three replicates were performed for each assay to ensure reproducibility. The primary data are shown in **Figure S22**.

As a control, we conducted a test assay with the commercial DPP-4 probe GP-AMC (H-Gly-Pro-AMC; Anaspec AS-24098) at the same reaction volume and final concentrations 25 µL of 10 µM probe (final probe concentration of 5 µM) and 25 µL human saliva in DPBS buffer and pH 7.6 at 27 °C (**Figure S21**). The assay with the commercial probe GP-AMC was monitored on the plate reader at the excitation wavelength of 380 nm and emission wavelength of 460 nm.

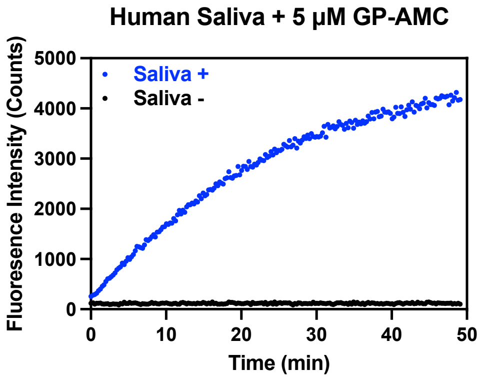

Figure S21. Commercial probe GP-AMC Cleaved in the Presence of Human Saliva. The assays were done with the saliva (+) and without the saliva (negative control; -). Fluorescence intensity was measured at 460 nm. All traces show the average of three replicates.

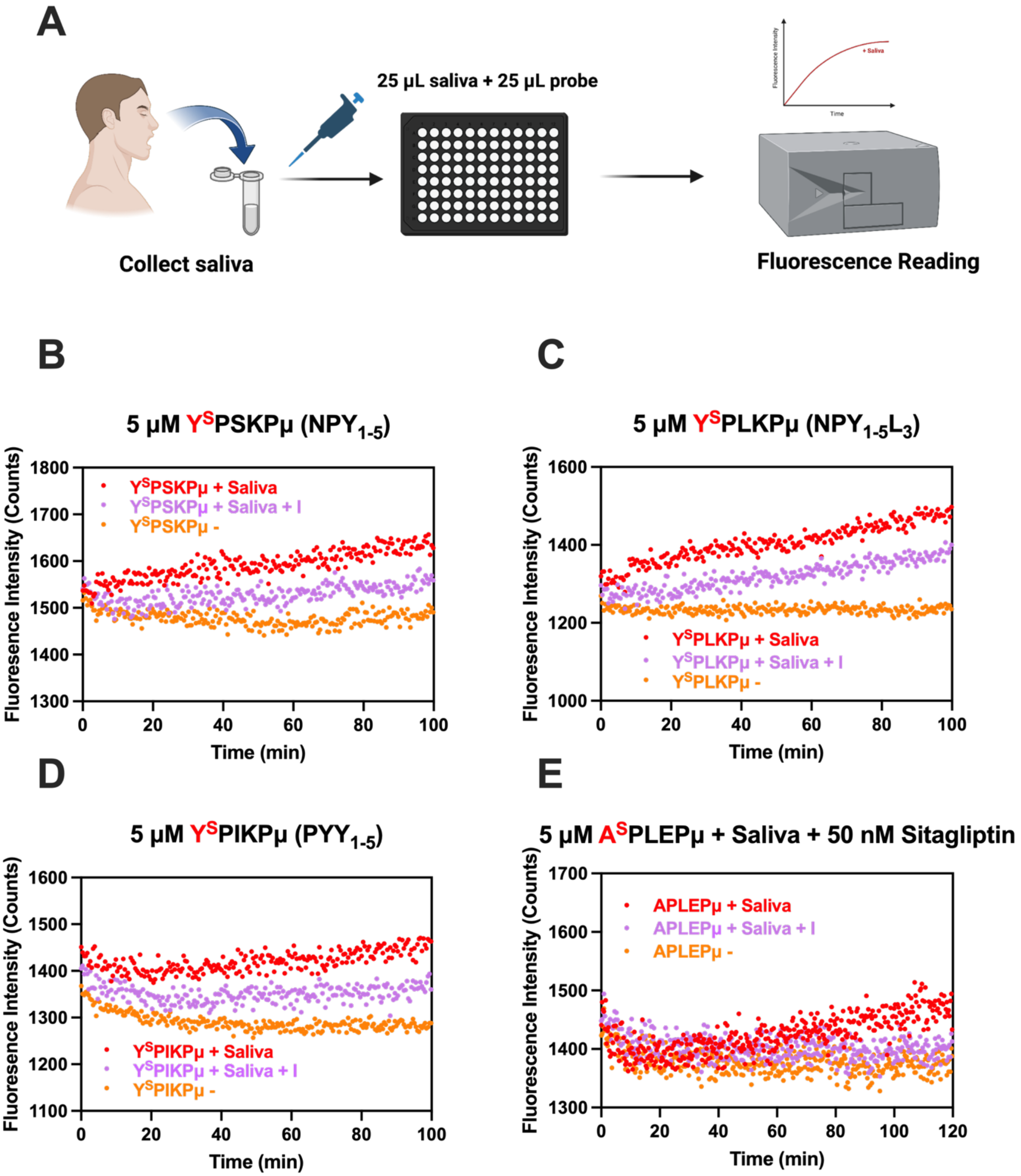

Figure S22. DPP-4 detection in human saliva. (A) Work-flow of the saliva assay. Saliva assay with the µ-tagged probes: (B) NPY-based Probes; (C) NPY-based Probes (P1'mutated); (D) PYY-based probes; (E) PP-based probes. For each assay, 25 µL of human saliva was added to 25 µL of 5 µM probe diluted in DPBS. The (-) indicates in the absence of the protease and the (+) indicates the presence of the protease. All traces show the average of three replicates. Figure (A) is created with BioRender.com.

7. DPP-4 Inhibition with Sitagliptin in Human Saliva

All assays were conducted with pooled human saliva commercially purchased from Innovative Research (Catalog #IRHUSL5ML). This experiment was done only with thioamide probes. For each probe, 25 µL of human saliva was added to 25 µL of 10 µM probe to a final probe concentration of 5 µM, except for the PP-based probes (15 µM). The probes were diluted in DPBS pH 7.6, and the assays were conducted at 27°C. The same protocol as the above human saliva assays was used, except that the saliva was pre-incubated either with or without sitagliptin (DPP-4 specific inhibitor, ≥98% by HPLC; Sigma-Aldrich SML3205) for 10 minutes before being added to the well containing the probe for the reactions. Sitagliptin is a DPP-4 inhibitor and has IC_{50} value of 18 nM. The final concentration of the inhibitor in the assays were 50 nM. That data for all the probes were shown in **Figure S23**.

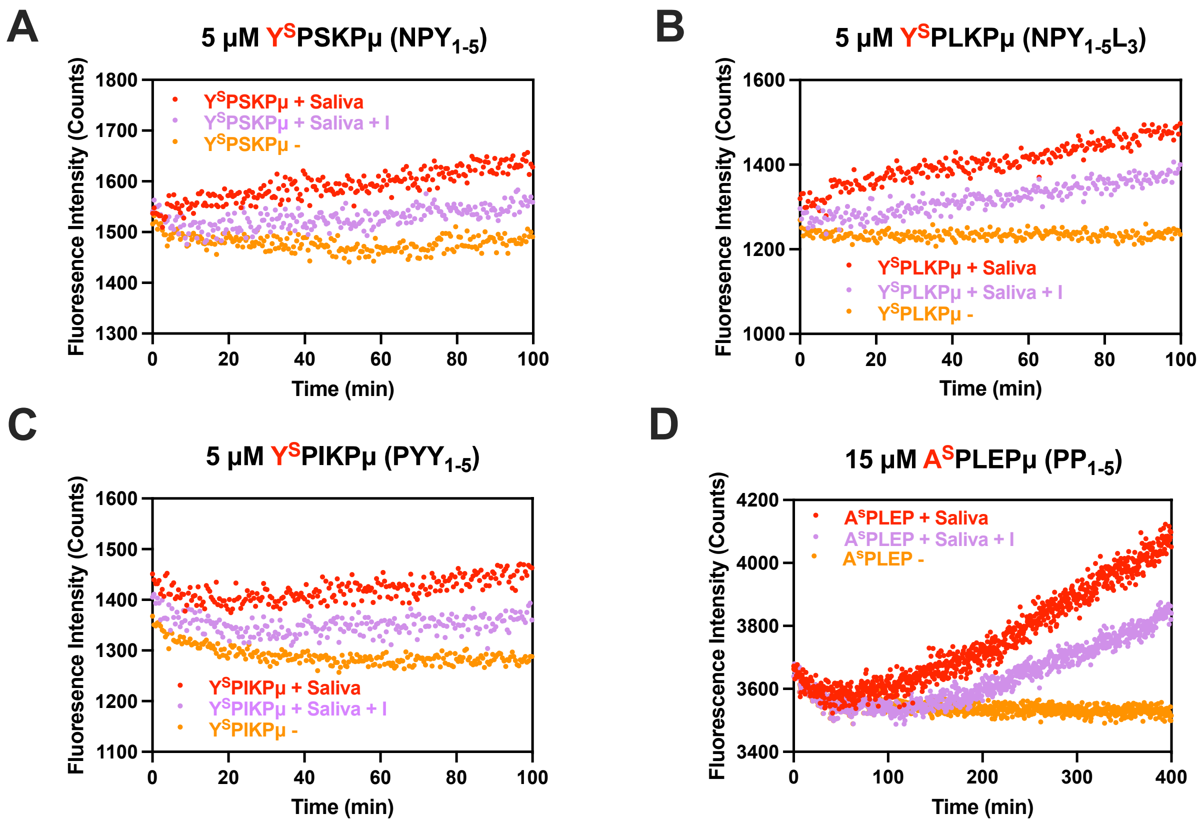

Figure S23. DPP-4 Inhibition by Sitagliptin Detected by Our Thioamide Probes in Human Saliva. For each protease, the assays were done with the protease (+), with the protease & 50 nM of the inhibitor (+ I), and without both the protease and inhibitor (negative control; -). Fluorescence intensity was measured at 325 nm. The fluorescence traces are shown for the probes: (A) NPY-based Probes (Y^SPSKPμ); (B) NPY-based Probes - P1'mutated (Y^SPLKPμ); (C) PYY-based probes (Y^SPIKPμ); (D) PP-based probe (A^SPLEPμ). All probes showed inhibition by sitagliptin, a specific inhibitor of DPP-4. All traces show the average of three replicates.

8. Human Saliva Doped with Additional DPP-4

To mimic diseased state biological samples that exhibit elevated DPP-4 level compared to samples from healthy donors, we conducted the assays where human saliva was doped with additional DPP-4 and validated whether if we could discern DPP-4 increase with our probes. All assays were conducted with pooled human saliva commercially purchased from Innovative Research (Catalog #IRHUSL5ML). This experiment was done only with NPY and PP-based thioamide probes. For each probe, 25 μL of human saliva was added to 25 μL of 10 μM NPY-based probe (Y^SPSKPμ) to a final probe concentration of 5 μM, and 30 μM PP-based probe (A^SPLEPμ) to a final probe concentration of 15 μM. The probes were diluted in DPBS pH 7.6, and the assays were conducted at 27°C. The same protocol as the above human saliva assays was used, except that additional two different concentrations of DPP-4 were added to the human saliva (the final concentrations of the DPP-4 were 500 ng/mL and 250 ng/mL). In addition, human saliva was pre-incubated either with or without sitagliptin (DPP-4 specific inhibitor, ≥98% by HPLC; Sigma-Aldrich SML3205) for 10 minutes before being added to the well containing the probe for the reactions. The final concentration of the inhibitor in the assays was 500 nM. The data for all the probes were shown in **Figure S24**.

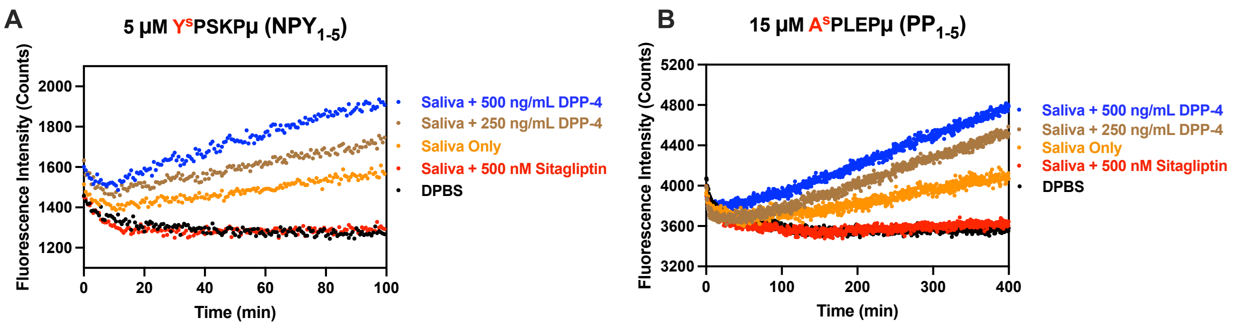

Figure S24. Measurement of DPP-4 level in human saliva with thioamide NPY_{1-5} and PP_{1-5} based probes. (A) Thioamide NPY-based probe (Y^SPSKPμ) or (B) Thioamide PP-based probe (A^SPLEPμ) was incubated with human saliva in the absence of or in the presence of additional DPP-4. In the presence of DPP-4 specific inhibitor sitagliptin, there was inhibition of the signal, indicating the specificity of our probe.

9. Nuclear Magnetic Resonance (NMR)

To gather more insight regarding the structural characteristic of our probe, we collected the following NMR spectra of the NPY-based all-amide peptide (YPSKPμ): COSY (Correlated Spectroscopy) (**Figure S25**), TOCSY (Total Correlation Spectroscopy) (**Figure S26**), and NOESY (Nuclear Overhayser Effect Spectroscopy) (**Figure S27**). The peptide sample was collected at 5 mM of peptide in 90%H_2O/10%D_2O with salt, pH = 6.5 (137 mM NaCl, 8.1 mM Na_2HPO_4, 2.7 mM KCl, and 1.5 mM KH_2PO_4). All spectra were collected with the AVANCE NEO 600 MHz NMR (Bruker). Our results are consistent with previous reports indicating that the C-terminus of NPY is unstructured.[4]

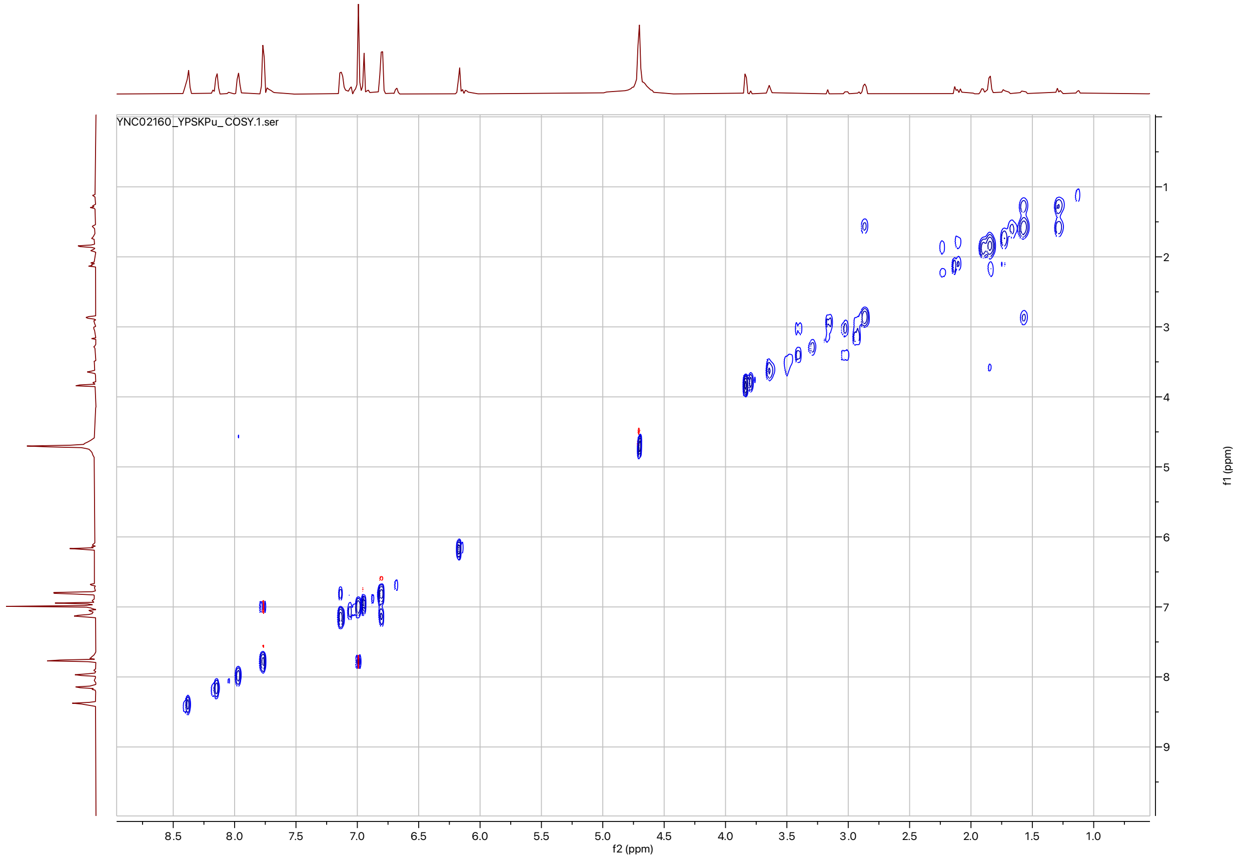

Figure S25. COSY of NPY-based peptide probe (YPSKPμ).

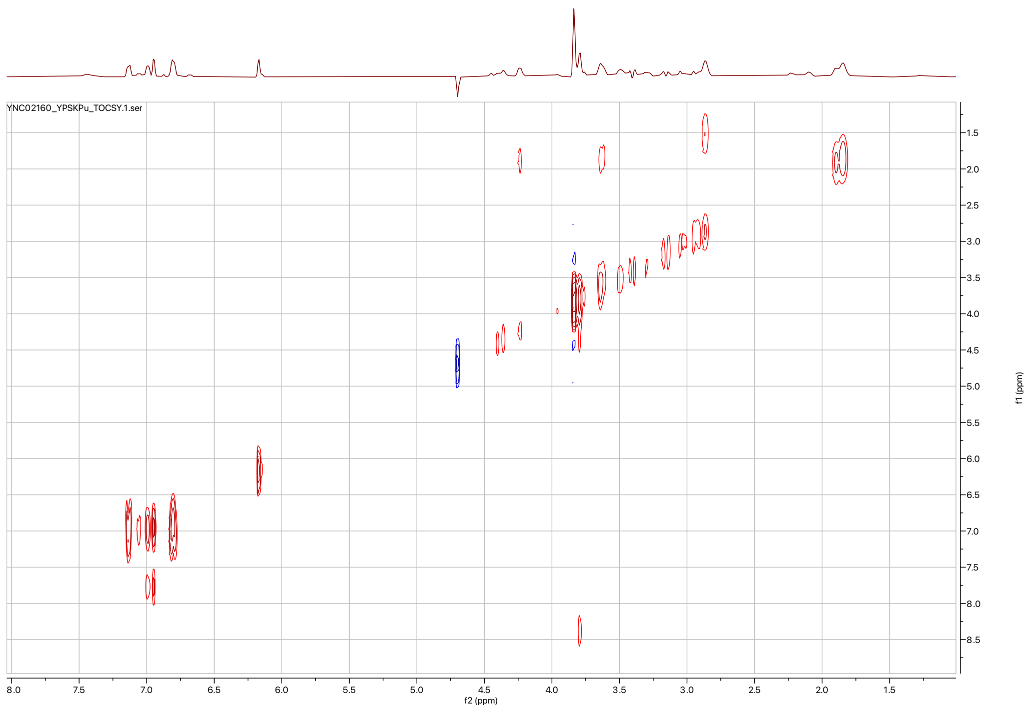

Figure S26. TOCSY of NPY-based peptide probe (YPSKPμ).

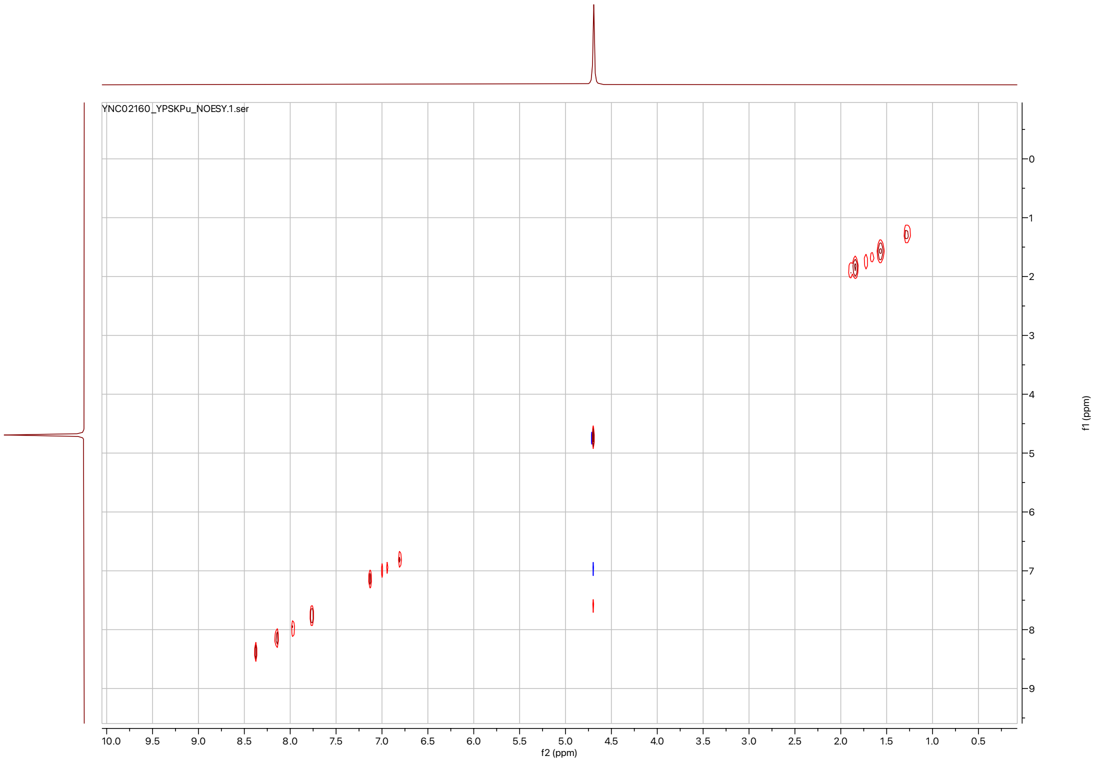

Figure S27. NOESY of NPY-based peptide probe (YPSKPμ).

10. References

1. T. M. Barrett, X. S. Chen, C. Liu, S. Giannakoulias, H. A. T. Phan, J. Wang, E. K. Keenan, R. J. Karpowicz, Jr. and E. J. Petersson, *ACS Chem Biol*, 2020, **15**, 774-779.
2. C. Liu, T. M. Barrett, X. Chen, J. J. Ferrie and E. J. Petersson, *ChemBioChem*, 2019, **20**, 2059-2062.
3. D. M. Szantai-Kis, C. R. Walters, T. M. Barrett, E. M. Hoang and E. J. Petersson, *Synlett*, 2017, **28**, 1789-1794.
4. V. Saudek and J. T. Pelton, *Biochemistry*, 1990, **29**, 4509-4515.
